# Geographical and Climatic Effects on the Quality Attributes of Chinese Mango Fruit

**DOI:** 10.3390/foods15193425

**Published:** 2026-09-24

**Authors:** Lihong Xie, Xuemei He, Youxia Shan, Ping Yi, Chuanyan Rao, Jinfeng Sheng, Ying Yang, Min Huang, Ting Gan, Dongqing Ye, Ming Xin, Xuefeng Wang, Zhichun Li, Li Li, Jian Sun

**Affiliations:** 1 Guangxi Key Laboratory of Fruits and Vegetables Storage-Processing Technology, Guangxi Academy of Agricultural Sciences, Nanning 530007, China; lihong@gxaas.net (L.X.); xuemeihegx@gxaas.net (X.H.); pingyi@gxaas.net (P.Y.); raochuanyan@gxaas.net (C.R.); shengjinfeng@gxaas.net (J.S.); yangying@gxaas.net (Y.Y.); hmin@gxaas.net (M.H.); ganting@gxaas.net (T.G.); yedongqing@gxaas.net (D.Y.); xinming-17@163.com (M.X.); xfw@gxaas.net (X.W.); lizhichun@gxaas.net (Z.L.); 2 Key Laboratory of National Forestry and Grassland Administration on Ecosystem Conservation and Restoration in the Guangdong-Hong Kong-Macao Greater Bay Area, Guangdong Provincial Key Laboratory of Forest Silviculture, Protection and Utilization, Guangdong Academy of Forestry, Guangzhou 510520, China; shanyouxia@126.com

**Keywords:** cultivar-specific correlation, fruit quality, geographic factors, mango, multivariate analysis

## Abstract

Mango (*Mangifera indica* L.) is a commercially important fruit crop whose quality attributes are shaped by genetic background and cultivation environment, yet the relative contributions of geographic origin, climatic factors, and cultivar remain poorly resolved across China’s diverse production regions. Forty mango groups representing six major cultivars (*Tainong No. 1*, *Jinhuang*, *Guifei*, *Guire 82*, *Keitt*, and *Eagle-mouth*) were collected from four principal production provinces (Guangxi, Hainan, Sichuan, and Yunnan), spanning latitudes from 19° N to 27° N and elevations from 6 to 1668 masl. Twelve quality traits were quantified alongside nine environmental variables. PCA captured 41.2% of total variance but revealed substantial interprovincial overlap, indicating limited discriminatory power of geographic origin alone. OPLS-DA showed that mean relative humidity (VIP = 1.48) and extreme maximum temperature (VIP = 1.15) emerged as the primary environmental discriminators among the variables examined. Hierarchical clustering revealed that samples from the same province were frequently dispersed across multiple clusters, further highlighting the apparent role of cultivar-specific genetic factors. Pooled Pearson correlation analysis showed that fruit-quality variation was associated mainly with cultivar: cultivar correlated significantly with total soluble solids (r = 0.598), carotene (r = 0.535), and pectin (r = 0.525), whereas environment–quality associations were weak and exploratory, with only two nominally significant correlations (extreme maximum temperature with carotene, r = −0.332, and with moisture, r = 0.327). These findings suggest that mango fruit quality is governed predominantly by cultivar, with local microclimate and geographic provenance serving as modulating factors. Such insights offer preliminary evidence-based guidance for regional cultivar deployment and quality-oriented orchard management, particularly in cultivar selection for targeted fresh-market and processing applications.

## 1. Introduction

Mango (*Mangifera indica* L.) is one of the most economically important fruit crops in tropical and subtropical regions worldwide, with global production surpassing 57 million tonnes in 2023 [1,2]. Beyond its economic significance, mango is highly valued for its distinctive flavor, aroma, and rich nutritional profile, which encompasses dietary fiber, carotenoids (particularly β-carotene and lutein), ascorbic acid, polyphenols, and a diverse array of volatile organic compounds (VOCs) that contribute to its unique sensory attributes [3,4]. In recent years, rising consumer awareness of health benefits associated with fruit consumption has intensified research efforts to understand the factors determining fruit quality, including total soluble solids content, sugar composition, organic acid profile, carotenoid accumulation, and textural properties [4,5,6,7,8]. To address this need, this study aims to quantify the relative contributions of cultivar and environment to quality traits, thereby providing a scientific basis for cultivar selection and region-specific cultivation practices to enhance fruit quality.

China has emerged as the world’s second-largest mango producer, with an annual output exceeding 3.5 million tonnes, ranking only behind India [1,9]. The country’s cultivation is uniquely characterized by remarkable geographic and climatic diversity, spanning tropical Hainan Island (18–20° N), subtropical Guangxi (22–24° N), and the high-altitude dry–hot valleys of Sichuan and Yunnan (26–27° N, 1000–1700 masl) [10,11]. This latitudinal and elevational gradient constitutes a natural laboratory for examining how climatic variables, including temperature regimes, precipitation patterns, relative humidity, and solar radiation, influence fruit developmental processes and ultimate quality outcomes. Notably, recent evidence suggests that altitudinal variation significantly affects morpho-physiological traits [10]. However, its influence on fruit quality and volatile profiles across China’s diverse mango growing regions remains poorly understood.

The relationship between environmental factors and fruit quality has been extensively investigated in horticultural science [12,13]. Temperature is widely recognized as a primary determinant of fruit developmental rate, sugar accumulation, organic acid metabolism, and secondary metabolite biosynthesis [14,15]. Elevated temperatures during fruit development have been associated with improved carotenoid biosynthesis and peel coloration [16]. However, excessive heat stress can suppress carotenoid biosynthesis while activating defense responses (e.g., elevated flavonoids and phenolic acids), ultimately compromising fruit quality [17]. Precipitation and irrigation regimes influence fruit turgor, cell expansion, and the dilution of soluble solids, while relative humidity affects transpiration rates, cuticular wax deposition, and susceptibility to physiological disorders [14]. Elevation, through its coupled effects on temperature, ultraviolet radiation, and diurnal thermal amplitude, modulates the accumulation of photoprotective secondary metabolites, including carotenoids and phenolic compounds [10,18,19]. Moreover, recent advances have revealed that low temperature can transcriptionally regulate sugar transporter genes to coordinate sugar accumulation in fruit, providing molecular-level insights into environment–quality relationships [20].

Despite this wealth of knowledge, several critical knowledge gaps persist. First, most prior studies examined individual environmental factors in isolation or focused on a single cultivar, limiting the ability to dissect the interactive effects of multiple climatic variables, geographic provenance, and cultivar-specific genetic programs [21,22]. Second, the relative importance of geographic origin versus cultivar in determining quality remains contentious. While some studies have reported significant inter-regional differences in fruit attributes [11], others have emphasized the predominance of genetic factors in shaping compositional profiles [3]. Third, the specific climatic drivers of quality differentiation within China’s diverse production landscape have not been systematically identified using multivariate chemometric approaches. Disentangling these contributions requires a systematic multi-region, multi-cultivar study design coupled with robust multivariate analytical tools.

Recent methodological advances offer powerful frameworks for addressing these challenges. Principal component analysis (PCA) and hierarchical clustering enable unbiased visualization of quality data structure and sample grouping patterns [23,24,25,26]. Orthogonal partial least squares discriminant analysis (OPLS-DA) and variable importance in projection (VIP) score were applied to select the most important variables for distinguishing our samples from different geographical origins, as previously reported [27,28]. Furthermore, cultivar-specific correlation network analysis offers a nuanced strategy to elucidate how individual cultivars respond to environmental gradients, thereby revealing genotype × environment (G × E) interaction patterns that are critical for regional cultivar deployment [23,29]. The G × E concept, well-established in perennial fruit crops, recognizes that phenotypic plasticity allows cultivars to exhibit differential metabolic responses to varying growing conditions, a phenomenon with direct implications for orchard management and quality optimization [30,31]. However, the integrated application of these multivariate tools to assess G × E interactions in late-maturing mango cultivars remains underexplored, warranting the present investigation.

In China, the mango cultivars *Tainong No. 1*, *Jinhuang*, *Guifei*, *Guire 82*, *Keitt*, and *Eagle-mouth* represent the major commercially grown varieties, each possessing distinct agronomic traits and quality characteristics [3,11]. Despite their commercial importance, systematic studies examining how these cultivars respond to the diverse environmental conditions across China’s production regions remain scarce. Therefore, the specific objectives of the present study were to (i) comprehensively characterize the twelve quality traits physicochemical profiles (including dietary fiber fractions, soluble sugars, titratable acids, carotene, pectin, and crude protein) of the six major cultivars sourced from four principal production provinces spanning China’s climatic gradient; (ii) employ multivariate statistical approaches (PCA, hierarchical clustering, and OPLS-DA with VIP scoring) to identify the key environmental determinants of quality variation and evaluate the discriminatory power of geographic origin versus cultivar; (iii) construct cultivar-specific environment–quality correlation networks to dissect G × E interaction signatures across representative cultivars; and (iv) provide evidence-based recommendations for cultivar-specific cultivation management and regional adaptation strategies that align with the emerging paradigm of terroir-driven fruit quality optimization. This integrated approach bridges a critical knowledge gap in the Chinese mango industry and offers a methodological framework applicable to other horticultural crops facing similar challenges of geographic and genetic complexity.

## 2. Materials and Methods

### 2.1. Reagents

Galacturonic acid, fructose, glucose, and sucrose reference standards were purchased from Sigma-Aldrich (St. Louis, MO, USA). HPLC-grade solvents were obtained from ANPEL Laboratory Technologies Inc. (Shanghai, China). Ethanol, hydrochloric acid, sulfuric acid, sodium hydroxide (NaOH), α-Naphthol, carbazole, butylated hydroxytoluene, and other analytical grade reagents were purchased from Xilong Scientific Co., Ltd. (Guangzhou, China). α-Amylase (A3403-500 KU from *Bacillus licheniformis*), protease (P0029 from *Bacillus* sp.), amyloglucosidase (A7095 from *Aspergillus niger*), and other enzyme preparations were purchased from Merck (Darmstadt, Germany).

### 2.2. Plant Materials and Growth Conditions

Mango fruits were collected from commercial orchards located in the four primary mango-producing provinces of China: Guangxi (n = 15), Hainan (n = 10), Sichuan (n = 9), and Yunnan (n = 6). Geographical and climatic data for the sampling sites were obtained from the China Meteorological Administration (CMA) (http://data.cma.cn, accessed on 22 September 2026). Data were matched to fruit samples by identifying the nearest CMA meteorological station for each sampling location based on city name, ensuring that the variables reflect local environmental conditions. The climatic data represent 30-year long-term averages from 1991 to 2020. The following environmental variables were extracted: latitude, longitude, elevation, mean annual temperature (MAT), extreme maximum temperature (T_max_), extreme minimum temperature (T_min_), temperature difference (ΔT = T_max_ − T_min_), annual precipitation (AP), and mean relative humidity (RH) (Table 1).

A total of 40 experimental groups were defined according to cultivar × origin combinations (Table 1). The collected samples comprised six major cultivars, including *Tainong No. 1* (n = 11), *Jinhuang* (n = 10), *Guifei* (n = 10), *Guire* 82 (n = 3), *Eagle-mouth* (n = 3), and *Keitt* (n = 3). For each group, 30 fruits were randomly divided into three independent biological replicates, each containing 10 fruits. Fruits were harvested at commercial maturity and then ripened with 500 μL L^−1^ ethylene gas until fully ripe and softened [32]. Samples were peeled, pitted, and diced into uniform fragments. The fruit fragments were immediately snap-frozen in liquid nitrogen. The resulting sample was stored at −80 °C for the analysis of twelve physicochemical traits. The measured parameters included total dietary fiber (TDF), insoluble dietary fiber (IDF), crude protein, moisture, carotene, total soluble solids, total acidity (TA), TSS/TA ratio, fructose, glucose, sucrose, and pectin.

### 2.3. Total Dietary Fiber, Insoluble Dietary Fiber and Crude Protein Content Analysis

Total dietary fiber (TDF), insoluble dietary fiber (IDF) and crude protein content were measured according to the enzymatic–gravimetric method [33], with slight modifications. A total of 650 g of the pulp underwent homogenization and was then rinsed with an 85% ethanol solution at a ratio of 10 mL per gram of sample. Following the desugaring process, the sample was air-dried overnight in an oven maintained at 40 °C, and the mass loss factor after desugaring and drying was recorded. The sample (0.5 g) of desugared and dried material was subsequently treated with enzymatic hydrolysis utilizing α-amylase, protease, and amyloglucosidase. The resultant hydrolysate was directly filtered, and the remaining solids were rinsed with 60 °C hot water. The non-digestible filter residue from the hydrolysate was gathered, washed, dried, and weighed to determine the mass of the IDF residue. Another 0.5 g portion of the desugared and dried sample experienced enzymatic hydrolysis, followed by the precipitation of the hydrolysate using 95% ethanol reagent (60 °C) for 1 h. This precipitate was collected, washed, dried, and weighed to ascertain the mass of the sample residue. The TDF and IDF residue masses were adjusted by deducting the remaining protein, ash, and reagent blank weights to yield the corresponding dietary fiber content. The measured protein and ash weights were deducted from the total weight to yield the net dietary fiber content via gravimetric analysis. The nitrogen (N) content of the hydrolysate was measured by Kjeldahl method, and the crude protein content (%) was derived using a conversion factor of 6.25. Ash content was determined by ashing at 525 °C for 5 h, followed by the calculation of the ash mass. The TDF and IDF content, Crude protein content was expressed as g/100 g and % of fresh weight (FW), respectively.

**Table 1:** Sample information and geographical/climatic characteristics of the mango fruit.

No.	Sample ID	Cultivar	Place	Latitude (°N)	Longitude (°E)	Elevation (masl)	MAT (°C)	T_max_ (°C)	T_min_ (°C)	ΔT (°C)	AP (mm)	RH (%)
1	TN1-GX	Tainong No.1	Tiandong, Baise, Guangxi	23.90	106.60	175	22.4	40.4	−0.2	40.6	1204	78
2	TN2-GX	Tainong No.1	Tiandong, Baise, Guangxi	23.90	106.60	175	22.4	40.4	−0.2	40.6	1204	78
3	TN3-GX	Tainong No.1	Youjiang, Baise, Guangxi	23.60	107.23	155	22.3	42.2	0.3	41.9	1104	76
4	TN4-GX	Tainong No.1	Tianyang, Baise, Guangxi	23.75	106.93	133	23.1	40.9	3.2	37.7	1112	75
5	TN5-HN	Tainong No.1	Changjiang, Hainan	19.43	109.08	98	25.2	40.5	5.4	35.1	1748	75
6	TN6-HN	Tainong No.1	Ledong, Hainan	18.40	108.65	98	24.8	33.0	15.8	17.2	1402	79
7	TN7-HN	Tainong No.1	Dongfang, Hainan	19.10	108.62	6	25.5	36.5	6.4	30.1	1042	78
8	TN8-SC	Tainong No.1	Yanbian, Panzhihua, Sichuan	27.15	102.42	1140	20.1	40.2	0.4	39.8	933	61
9	TN9-YN	Tainong No.1	Baoshan, Yunnan	25.12	99.18	1668	16.8	32.4	−3.8	36.2	964	70
10	TN10-YN	Tainong No.1	Baoshan, Yunnan	25.12	99.18	1668	16.8	32.4	−3.8	36.2	964	70
11	TN11-YN	Tainong No.1	Lincang, Yunnan	23.88	100.08	1636	18.1	34.4	−1.0	35.4	1126	70
12	JH1-GX	Jinhuang	Youjiang, Baise, Guangxi	23.60	107.23	155	22.3	42.2	0.3	41.9	1104	76
13	JH2-GX	Jinhuang	Youjiang, Baise, Guangxi	23.60	107.23	155	22.3	42.2	0.3	41.9	1104	76
14	JH3-GX	Jinhuang	Tiandong, Baise, Guangxi	23.90	106.60	175	22.4	40.4	−0.2	40.6	1204	78
15	JH4-GX	Jinhuang	Tianyang, Baise, Guangxi	23.75	106.93	133	23.1	40.9	3.2	37.7	1112	75
16	JH5-HN	Jinhuang	Changjiang, Hainan	19.43	109.08	98	25.2	40.5	5.4	35.1	1748	75
17	JH6-HN	Jinhuang	Dongfang, Hainan	19.10	108.62	6	25.5	36.5	6.4	30.1	1042	78
18	JH7-HN	Jinhuang	Ledong, Hainan	18.40	108.65	98	24.8	33.0	15.8	17.2	1402	79
19	JH8-SC	Jinhuang	Miyi, Panzhihua, Sichuan	27.53	102.20	1137	20.0	40.3	−1.9	42.2	1070	64
20	JH9-SC	Jinhuang	Miyi, Panzhihua, Sichuan	27.53	102.20	1137	20.0	40.3	−1.9	42.2	1070	64
21	JH10-SC	Jinhuang	Yanbian, Panzhihua, Sichuan	27.15	102.42	1140	20.1	40.2	0.4	39.8	933	61
22	GF1-GX	Guifei	Youjiang, Baise, Guangxi	23.60	107.23	155	22.3	42.2	0.3	41.9	1104	76
23	GF2-GX	Guifei	Youjiang, Baise, Guangxi	23.60	107.23	155	22.3	42.2	0.3	41.9	1104	76
24	GF3-GX	Guifei	Tiandong, Baise, Guangxi	23.90	106.60	175	22.4	40.4	−0.2	40.6	1204	78
25	GF4-GX	Guifei	Tianyang, Baise, Guangxi	23.75	106.93	133	23.1	40.9	3.2	37.7	1112	75
26	GF5-HN	Guifei	Changjiang, Hainan	19.43	109.08	98	25.2	40.5	5.4	35.1	1748	75
27	GF6-HN	Guifei	Changjiang, Hainan	19.43	109.08	98	25.2	40.5	5.4	35.1	1748	75
28	GF7-HN	Guifei	Ledong, Hainan	18.40	108.65	98	24.8	33.0	15.8	17.2	1402	79
29	GF8-HN	Guifei	Dongfang, Hainan	19.10	108.62	6	25.5	36.5	6.4	30.1	1042	78
30	GF9-SC	Guifei	Miyi, Panzhihua, Sichuan	27.53	102.20	1137	20.0	40.3	−1.9	42.2	1070	64
31	GF10-SC	Guifei	Miyi, Panzhihua, Sichuan	27.53	102.20	1137	20.0	40.3	−1.9	42.2	1070	64
32	GR1-GX	Guire 82	Youjiang, Baise, Guangxi	23.60	107.23	155	22.3	42.2	0.3	41.9	1104	76
33	GR2-GX	Guire 82	Youjiang, Baise, Guangxi	23.60	107.23	155	22.3	42.2	0.3	41.9	1104	76
34	GR3-GX	Guire 82	Tiandong, Baise, Guangxi	23.90	106.60	175	22.4	40.4	−0.2	40.6	1204	78
35	EM1-YN	Eagle-mouth	Baoshan, Yunnan	25.12	99.18	1668	16.8	32.4	−3.8	36.2	964	70
36	EM2-YN	Eagle-mouth	Baoshan, Yunnan	25.12	99.18	1668	16.8	32.4	−3.8	36.2	964	70
37	EM3-YN	Eagle-mouth	Yuanjiang, Yunnan	23.60	101.98	507	24.1	42.5	−0.1	42.6	802	71
38	KT1-SC	Keitt	Miyi, Panzhihua, Sichuan	27.53	102.20	1137	20.0	40.3	−1.9	42.2	1070	64
39	KT2-SC	Keitt	Miyi, Panzhihua, Sichuan	27.53	102.20	1137	20.0	40.3	−1.9	42.2	1070	64
40	KT3-SC	Keitt	Yanbian, Panzhihua, Sichuan	27.15	102.42	1140	20.1	40.2	0.4	39.8	933	61

Note: Abbreviations for the six cultivars: *EM*, *Eagle-mouth*; *GF*, *Guifei*; *GR*, *Guire 82*; *JH*, *Jinhuang*; *KT*, *Keitt*; *and TN*, *Tainong No*. *1*. The suffix of group ID denotes the province of origin: GX, Guangxi; HN, Hainan; SC, Sichuan; YN, Yunnan. The Sample ID follows the naming convention: Cultivar abbreviation—sequential number of the independent experimental group—province abbreviation. MAT, mean annual temperature; T_max_, extreme maximum temperature; T_min_, extreme minimum temperature; ΔT, temperature difference (T_max_ − T_min_); AP, annual precipitation; RH, mean relative humidity.

### 2.4. Moisture Contents Analysis

The moisture content of the pulp was determined using the direct drying method. The homogenized the pulp sample (5.0 g) was accurately weighed and placed into a pre-weighed aluminum weighing dish. The samples were then dried in an air oven at 105 °C until a constant weight was attained. The moisture content was calculated based on the weight loss of sample and expressed as g/100 g.

### 2.5. Pectin Contents Analysis

Pectin was determined using the sulfuric acid-carbazole colorimetric method, adhering to the procedures outlined in the Chinese agricultural standard NY/T 2016-2011 [34,35], with minor modifications. In brief, 1.0 g of pulp powder was combined with 35 mL anhydrous ethanol and heated at 85 °C for 10 min with thorough vortexing. After cooling, the solution was adjusted to a final volume of 50 mL with anhydrous ethanol and centrifuged at 4000 rpm for 15 min. The supernatant was discarded to eliminate soluble sugars and pigments. The precipitate was washed repeatedly with preheated anhydrous ethanol (85 °C) until the pellet turned white and tested negative with the α-naphthol test, confirming the complete removal of sugars. For acid extraction, the washed precipitate was transferred to a centrifuge tube containing sulfuric acid solution (pH 0.5), homogenized, and incubated at 85 °C for 60 min. After cooling, the extract was diluted to 100 mL with sulfuric acid solution (pH 0.5). An aliquot of 1.0 mL of the diluted extract was mixed with 0.25 mL carbazole ethanol solution (1 g/L) and subjected to color development following the same protocol as the standard series. The absorbance of reaction mixture was measured at 525 nm using a microplate reader (Synergy H1, BioTek Instruments, Winooski, VT, USA). Galacturonic acid served as the external standard, and pectin content was calculated based on a standard calibration curve. The pectin content was expressed as g/100 g.

### 2.6. Carotene Content Analysis

The carotene content of pulp was quantified using a spectrophotometric method based on solvent extraction [36,37], with minor modifications. Briefly, pulp powder (1.0 g) was extracted with 30 mL of acetone containing 0.1% butylated hydroxytoluene. The mixture was vortexed and then ultrasonicated for 30 min. After filtration, the supernatant was collected. The residue was washed twice with 15 mL of acetone each time, and the filtrates were combined with the first supernatant. The pooled extracts were transferred to a separatory funnel, to which 20 mL of distilled water and 30 mL of petroleum ether were added to induce phase separation. After thorough mixing and standing, the upper organic layer was collected and dehydrated with 2 g of anhydrous sodium sulfate. The dried extract was filtered into a 50 mL brown volumetric flask and made up to volume with petroleum ether. The absorbance was measured using a UV-Vis spectrophotometer (UV 1800, Shanghai Meixi Instruments Co., Ltd., Shanghai, China), with petroleum ether as the blank. The carotene content (mg/100 g) was calculated using a standard curve of β-carotene.

### 2.7. Total Soluble Solids and Total Acid Content Analysis

Total soluble solids (TSS) content was determined with a handheld digital refractometer (PLA-BX/ACID15, ATAGO, Tokyo, Japan). Fresh fruits were peeled and pitted, followed by the homogenization of flesh. The homogenate was filtered to obtain the clarified juice. TSS content was measured using 1 mL of the undiluted juice. Total acid (TA) contents were measured using acid-base titration with 0.01 M NaOH, according to our previous method [38]. TA content was determined with 10.0 g of the clarified juice and diluted to 25.0 g using purified water. Based on the obtained TSS and TA values, the solid–acid ratio (TSS/TA) was subsequently calculated by dividing TSS by TA.

### 2.8. Soluble Sugars Content Analysis

Fructose, glucose, and sucrose content was analyzed using the pulp powder using high-performance liquid chromatography (HPLC) system (Shimadzu, Kyoto, Japan), according to our previous method [39]. Chromatographic data were acquired and processed using the Shimadzu Corporation LabSolutions V1.0.6653.30946 software. Briefly, a total of 2.0 g of pulp powder was combined with 10 mL of 80% (*v*/*v*) ethanol and incubated for 30 min at 80 °C in a water bath. This extraction process was carried out a second time, with the resulting supernatants collected via centrifugation at 10,000× *g* for 10 min at 4 °C. Following centrifugation, the supernatant was passed through a 0.45 μm membrane filter and subsequently purified using a C18 solid-phase extraction cartridge to eliminate pigments and proteins. The purified extract underwent analysis with HPLC utilizing a refractive index detector (RID). Chromatographic separation was performed using a Supersil NH2-S Plus amino column (5 μm; Dalian Elite Analytical Instruments Co., Ltd., Dalian, China). The presence of fructose, glucose, and sucrose was confirmed by comparing retention times to those of known standards, and quantification was achieved using external standard calibration curves. Soluble sugar contents were expressed as mg/g FW.

### 2.9. Statistical Analysis

All data were expressed as means ± standard errors (SEs) from three biological replicates. One-way ANOVA followed by Duncan’s multiple range test was conducted using R (v4.3.0) to identify significant differences among groups (*p* < 0.05). Multivariate analyses were performed entirely within the R environment. PCA was implemented via the prcomp function with data scaled and centered, and biplots and loading plots were generated using ggplot2 and ggrepel for dimensionality reduction and trait contribution interpretation. Hierarchical clustering was carried out with Ward’s linkage method based on the Euclidean distance of Z-score-normalized data using the pheatmap package. OPLS-DA was performed using the ropls package with three predictive components (predI = 3) under standard scaling, as configured in the ropls discriminant-analysis workflow used to generate the model, and VIP scores were calculated to rank environmental discriminators (VIP > 1.0 considered significant). The validity and performance of this supervised discriminant model were characterized by its goodness-of-fit R2Y(cum) (cumulative fraction of response variance explained) and its internally cross-validated predictive ability Q2(cum), together with a classification-confusion analysis; statistical significance was verified by class-label permutation testing (n = 200), from which the empirical pR2Y and pQ2 values were extracted (ropls output). Pearson correlation analyses were performed on the pooled dataset of all 40 samples using the rcorr function from the Hmisc package, with statistical significance set at *p* < 0.05 (nominal, unadjusted); in addition, Benjamini–Hochberg false discovery rate (FDR) correction was applied to evaluate the robustness of significant correlations to multiple testing. Correlation patterns were visualized as a single pooled chord diagram via the circlize package. All graphical outputs, including boxplots, heatmaps, and scatter plots, were generated using ggplot2 (v3.5.1), patchwork for plot assembly, and cowplot for figure arrangement. Data preprocessing and manipulation were executed using the dplyr, tidyr, and readxl packages. All R scripts and raw data are available upon request.

## 3. Results

### 3.1. Geographic Distribution, Clustering, and Heatmap Profiling of Mango Germplasm Quality Traits

To characterize the spatial distribution of mango genetic resources in China, sampling sites of six cultivars were mapped across four principal production provinces (Figure 1A). Representative fruit images illustrating morphological variation among the six cultivars data presented in Figure 1B, *Tainong No. 1* and *Guifei* jointly accounted for 52.5% of the total sample pool, whereas *Keitt*, *Guire 82*, and *Eagle-mouth* were represented by fewer accessions, reflecting their restricted cultivation scope or specialized market niches.

Hierarchical clustering based on the 12 measured physicochemical parameters (Ward’s method) was performed to resolve intrinsic relationships among the 40 accessions. The resultant dendrogram (Figure 1C) separated samples into three major clusters that exhibited limited concordance with provincial boundaries. Yunnan and Sichuan accessions tended to group on the upper branches, suggesting a degree of compositional similarity, while Guangxi and Hainan samples were distributed across all three clusters, indicating pronounced within-region heterogeneity. This pattern implies that cultivar and localized agronomic conditions exert stronger control over fruit quality than geographic origin alone. In addition, a heatmap constructed using Z-score-normalized values (0–10 scale) further delineated trait-level variation (Figure 1C). Soluble solids, fructose, sucrose, and the TSS/TA ratio were consistently elevated in Sichuan and Yunnan samples, whereas moisture and total dietary fiber were enriched in Guangxi and Hainan accessions. Cultivar-specific signatures were also discernible: *Keitt* accessions exhibited comparatively lower TSS and sucrose concentrations, while *Tainong No. 1* maintained intermediate and relatively stable profiles across multiple quality attributes. Collectively, the clustering and heatmap analyses reveal that the fruit quality is shaped by a complex interplay between genetic background and production environment.

### 3.2. Principal Component Analysis of Mango Quality Profiles Across Geographic Origins

PCA results were visualized through a biplot (Figure 2A), a scree plot (Figure 2B), and a loading plot (Figure 2C). The first two principal components (PCs) cumulatively explained 41.2% of total variance, with PC1 accounting for 23.7% and PC2 for 17.5%. The score plot (Figure 2A) demonstrated considerable overlap among samples from Guangxi, Hainan, and Sichuan, which occupied a broad central region (PC1 mean scores: 0.12 ± 1.77, −0.13 ± 1.97, and 0.65 ± 1.19, respectively), indicating the absence of distinct geographic clustering. Yunnan samples exhibited a modest displacement toward negative PC1 values (mean: −1.06 ± 1.39), suggestive of partial compositional differentiation. PC1 was predominantly driven by crude protein (loading = −0.483), moisture (0.452), total dietary fiber (−0.445), and carotene (−0.301), delineating a gradient between samples enriched in protein, fiber, and carotene (negative direction) and those characterized by elevated moisture and TSS/TA ratio (positive direction). PC2 was governed chiefly by the TSS/TA ratio (−0.508), total acidity (0.462), glucose (−0.417), and TSS (−0.283), capturing variation in sugar–acid balance. The scree plot confirmed that the first three PCs explained over 54% of cumulative variance, supporting retention of two components for interpretation. Collectively, PCA revealed largely overlapping quality profiles across provinces, with crude protein, moisture, dietary fiber, and sugar–acid equilibrium contributing to subtle regional differentiation. The pronounced within-province dispersion underscores that cultivar and localized agronomic practices exert a stronger influence on fruit quality than geographic origin alone.

### 3.3. Provincial-Level Variation in Fruit Quality Attributes

Twelve physicochemical traits were quantified across 40 fruit samples sourced from four principal production provinces in China (Figure 3). Dietary fiber fractions and proximate compositions are presented in Figure 3A–D. Mean TDF content exhibited a narrow range, from 1.34 ± 0.24 g/100 g in Guangxi to 1.50 ± 0.37 g/100 g in Hainan, with no significant variation detected among provinces (*p* = 0.621). IDF followed a comparable pattern, spanning 0.51 ± 0.23 g/100 g (Hainan) to 0.62 ± 0.21 g/100 g [40]. Crude protein remained relatively uniform (1.21–1.52 g/100 g), while moisture content was consistently high across all regions (84.6–86.5 g/100 g). None of these proximate constituents achieved statistical significance at the provincial level.

In addition, carotene concentrations (Figure 3E) demonstrated pronounced within-province dispersion, with Yunnan recording the highest mean (36.62 ± 18.94 mg/100 g) and Sichuan the lowest (15.92 ± 6.20 mg/100 g). Despite the considerably elevated median observed in Yunnan, the overall provincial effect failed to reach significance (*p* = 0.097), a result plausibly attributable to the limited sample size from Yunnan and substantial intra-group variance. Regarding soluble sugars and organic acid profiles (Figure 3F–L), TSS averaged between 14.7 and 18.5 °Brix without discernible geographic differentiation (*p* = 0.159). TA ranged from 3.00 ± 1.20 g/100 g in Hainan to 5.37 ± 1.81 g/100 g in Yunnan, while the TSS/TA ratio, an integrative sensory indicator, varied from 3.76 ± 1.36 (Yunnan) to 6.13 ± 3.06 (Hainan). Fructose was confirmed as the dominant monosaccharide across all provinces (4.6–5.0 g/100 g), followed by glucose (0.9–1.3 g/100 g). Sucrose, representing the most abundant individual sugar, ranged from 8.47 ± 2.21 g/100 g (Yunnan) to 12.15 ± 2.88 g/100 g (Sichuan), yet this apparent gradient did not attain statistical significance (*p* = 0.102). Pectin content remained uniformly low across samples (0.20–0.25 g/100 g).

Critically, one-way ANOVA detected no statistically significant differences among the four provinces for any of the twelve evaluated quality traits (all *p* > 0.05). Carotene content exhibited the largest inter-provincial disparity, approaching but not surpassing the conventional threshold (*p* = 0.097). These results demonstrate that provincial origin alone possesses limited discriminatory power for fruit quality within the present sampling design. Instead, the substantial within-province variability, arising from cultivar diversity, orchard-specific microclimate, harvest maturity stage, and postharvest management, appears to constitute the predominant source of quality variance. This comprehensive characterization of fruit physicochemical attributes reinforce the conclusion that genetic identity and localized agronomic factors exert a stronger influence on fruit composition than broad geographic provenance alone.

### 3.4. Environmental Drivers of Regional Fruit Quality Differentiation

Figure 4A–I summarizes the distribution of nine environmental variables, and Figure 4J displays their VIP scores from OPLS-DA. Pronounced inter-provincial divergence was evident for all parameters. Latitude extended from 19.02 ± 0.45° N in Hainan to 27.41 ± 0.19° N in Sichuan (Figure 4A)**.** Meanwhile, elevation exhibiting the greatest disparity from 6 to 1668 masl, with Yunnan recorded the highest mean altitude (1469.42 ± 471.76 masl), whereas Hainan (70.59 ± 44.50 masl) and Guangxi (157.29 ± 15.43 masl) occupied low-elevation terrains (Figure 4C). Thermal regimes varied substantially, with mean annual temperature (MAT) peaking in Hainan (25.20 ± 0.30 °C) and reaching a minimum in Yunnan (18.23 ± 2.90 °C). Diurnal temperature amplitude was notably larger in Sichuan (41.40 ± 1.20 °C) and Guangxi (40.63 ± 1.63 °C) relative to Hainan (28.23 ± 7.92 °C). Annual precipitation (AP) ranged from 964.13 ± 102.27 mm in Yunnan to 1432.42 ± 308.95 mm in Hainan, while mean relative humidity (RH) was highest in Hainan (77.10 ± 1.85%) and lowest in Sichuan (63.00 ± 1.50%) (Figure 4H,I).

**Figure 4:**
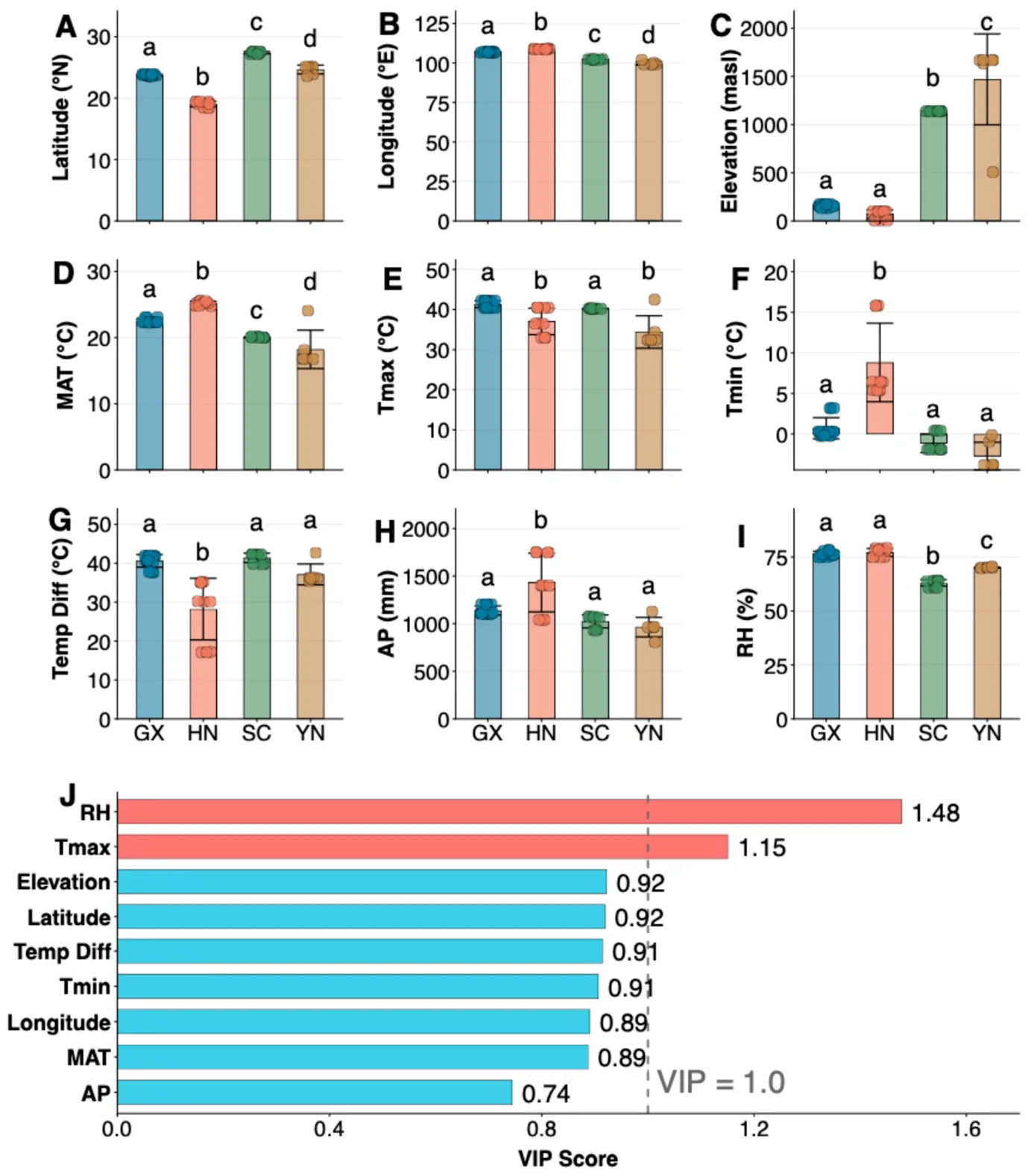
Comparison of environmental conditions across four growing provinces and VIP score ranking of the contributing factors. (**A**) Latitude; (**B**) Longitude; (**C**) Elevation; (**D**) MAT, mean annual temperature; (**E**) T_max_, extreme maximum temperature; (**F**) T_min_, extreme minimum temperature; (**G**) Temp Diff, temperature difference (T_max_ − T_min_); (**H**) AP, annual precipitation; (**I**) RH, mean relative humidity; (**J**) VIP scores.The upper panels A-I shows the distribution of six climatic and geographical variables across the four regions. The lower panels displays the VIP scores, with a threshold of 1.0 marked by a dashed line, indicating that RH, T_max_, T_min_, and Temp Diff (ΔT) were the most significant variables for region discrimination. Difference letters above the column represented the significant differences (*p* < 0.05).

The OPLS-DA model (Figure 4J) identified RH as the most influential discriminator (VIP = 1.48), followed by extreme maximum temperature (VIP = 1.15), both exceeding the threshold of 1.0. The validity and performance of this model were evaluated explicitly (Figure 4J). On the nine standardized environmental variables, the three-predictive-component discriminant model separated the four provinces with R2X(cum) = 0.921, R2Y(cum) = 0.798, Q2(cum) = 0.764 and RMSEE = 0.198. Class-label permutation testing (n = 200) confirmed that this discrimination was highly significant (pR2Y = 0.005, pQ2 = 0.005), and every permuted model returned a Q2 well below the observed value (permuted Q2 range: −0.448 to +0.030 vs. observed 0.764), demonstrating that the separation is robust and not an artefact of overfitting. Consistently high re-substitution classification accuracy (97.5%, 39/40 samples) was achieved, with only a single Yunnan sample misclassified as Guangxi. VIP ranking was therefore interpreted from this validated model. Complete validation and performance statistics are listed in Supplementary Tables S1 and S2. Elevation, latitude, temperature difference, and extreme minimum temperature contributed moderately (VIP 0.91–0.92), whereas longitude, mean annual temperature, and annual precipitation (VIP 0.74–0.89) exhibited weaker discriminatory capacity. These results establish RH and T_max_ as the primary climatic factors distinguishing mango-producing regions, with the markedly lower RH in Sichuan and contrasting thermal extremes between coastal and inland provinces likely imposing differential physiological pressures that shape fruit metabolic processes and regional quality attributes.

### 3.5. Cultivar- and Region-Dependent Variation in Fruit Quality Attributes

#### 3.5.1. Total Dietary Fiber and Insoluble Dietary Fiber

TDF and IDF contents differed substantially across cultivars and production regions. *Tainong No. 1* consistently recorded the highest mean values for both parameters, reflecting an inherently robust capacity for fiber accumulation. In contrast, *Jinhuang* and *Guifei* exhibited moderate and broadly equivalent fiber levels. The absolute range of TDF spanned from 0.7 g/100 g in Yunnan-sourced *Tainong No. 1* to 2.0 g/100 g in Hainan-sourced fruits of the same cultivar, demonstrating a pronounced environmental modulation of fiber biosynthesis. IDF, as the predominant fraction of TDF, displayed analogous spatial and varietal divergence (Appendix Figure A1E–H). The maximum IDF concentration (1.1 g/100 g) was observed in *Guifei* from Hainan, whereas the minimum (0.3 g/100 g) was shared by *Guifei* from Guangxi (GF3-GX) and specific Hainan-derived samples of *Tainong No. 1* and *Guifei*. Within *Tainong No. 1*, IDF peaked in the Guangxi accession TN3-GX (1.0 g/100 g). *Jinhuang* accessions JH1-GX [40] and JH10-SC (Sichuan) emerged as prominent outliers with markedly elevated IDF values. *Keitt* maintained a comparatively stable IDF profile (0.5–0.6 g/100 g). The coexistence of extreme IDF values among Hainan samples reveals a strong environmental regulation of insoluble fiber deposition that operates independently of TDF accumulation.

#### 3.5.2. Moisture and Pectin Content

Moisture content varied significantly among cultivars and production places, ranging from 78% (*Tainong No. 1*, Hainan) to 91% (*Tainong No. 1*, Guangxi; *Eagle-mouth*, Yunnan) (Figure A2A–D). Considerable intra-cultivar variability was observed, with *Jinhuang* accessions differing by up to nine percentage points between locations. Pectin content exhibited strong cultivar-specific accumulation patterns (Figure A2E–H). *Jinhuang* displayed the widest fluctuation, with Sichuan accessions differing nearly 2.5-fold (JH9-SC, 0.37 g/100 g versus JH10-SC, 0.15 g/100 g), implicating localized microenvironmental conditions in pectin biosynthesis. *Tainong No. 1* maintained a more balanced pectin distribution (mean 0.23 g/100 g), whereas *Guifei* consistently produced lower pectin levels (mean 0.16 g/100 g). Notably, the *Keitt* accession KT2-SC reached 0.36 g/100 g, considerably exceeding co-analyzed *Guire 82* and *Eagle-mouth* samples. These observations indicate that moisture and pectin accumulation are jointly governed by cultivar determinants and genotype-by-environment interactions, with pectin likely contributing to the water-holding capacity of the mesocarp.

#### 3.5.3. Crude Protein and Carotene Content

Crude protein content exhibited substantial phenotypic plasticity across growing environments (Figure A3A–D). *Tainong No. 1* reached 2.2% in Hainan (TN7-HN) yet only 1.0% in Guangxi (TN1-GX). *Guifei* similarly demonstrated regional superiority, with Hainan accession GF7-HN attaining 1.8%, outperforming *Guire 82* and *Keitt*, which generally remained below 1.5%. Carotene concentrations displayed even more dramatic environmental sensitivity (Figure A3E–H). *Tainong No. 1* varied approximately 4.7-fold between Sichuan (TN8-SC, 70 mg/100 g) and Yunnan (TN7-YN, 15 mg/100 g). *Guire 82* emerged as a high-potential cultivar for carotene enrichment, with the Guangxi accession GR2-GX reaching 65 mg/100 g, far surpassing *Eagle-mouth* and *Keitt* (20–30 mg/100 g). These pronounced disparities underscore the necessity of matching cultivars with optimal production regions for functional food applications.

#### 3.5.4. Total Soluble Solids, Acidity, and TSS/TA Ratio

TSS ranged from 13.1 °Brix (*Jinhuang*, Hainan) to 22.4 °Brix (*Tainong No. 1*, Yunnan). Guire 82 achieved particularly high TSS levels, with the Guangxi accession GR1-GX attaining 21.9 °Brix (Figure A4A–D). TA varied considerably, with Guire 82 (GR2-GX) reaching 10.4% and *Jinhuang* (JH6-HN) as low as 1.3% (Figure A4E–H). The TSS/TA ratio, reflecting overall flavor balance, exceeded 12 in select *Guifei* accessions from Guangxi, indicating a pronounced sweet-dominant profile, whereas *Keitt* consistently exhibited ratios between 2 and 5, characteristic of a more acidic sensory experience (Figure A4A–D). These patterns confirm that regional conditions strongly modulate flavor chemistry beyond baseline genetic effects.

#### 3.5.5. Fructose, Glucose, and Sucrose Content

Fructose, glucose and sucrose concentrations displayed marked genetic and geographic variation (Figure A5). *Tainong No. 1* fructose content ranged from 3.7 mg/g (Hainan) to 6.6 mg/g (Yunnan). *Guire 82* excelled in both sugars, with accession GR1-GX recording 5.5 mg/g fructose and 2.4 mg/g glucose, substantially exceeding *Keitt* and *Eagle-mouth*. Glucose exhibited acute within-cultivar sensitivity to microclimate; *Tainong No. 1* from Guangxi varied more than fivefold between accessions (TN4-GX, 2.1 mg/g versus TN1-GX, 0.4 mg/g). *Tainong No. 1* displayed moderate to high sucrose content, ranging from 7.8 mg/g (TN6-HN) to 12.2 mg/g (TN3-YN). In contrast, *Guire 82* showed consistently higher sucrose levels, with accession GR2-YN reaching 13.5 mg/g, the highest among all samples. *Keitt* and *Eagle-mouth* generally exhibited relatively low sucrose accumulation, with most accessions falling below 10 mg/g. Sucrose was the dominant soluble sugar in most samples, accounting for 50–70% of total soluble sugars. These findings confirm that while cultivar genetics establish sugar accumulation potential, the realization of high-sugar phenotypes depends critically on the cultivation environment, positioning Guangxi as a favorable region for maximizing sweetness-related quality traits.

Collectively, the quality profiles characterized in this section demonstrate that the nutritional and sensory attributes of mango are shaped by a complex interplay of cultivar, geographic provenance, and their interaction. Such multidimensional variability underscores the necessity of region-specific cultivar deployment strategies to optimize fruit quality for both fresh consumption and targeted processing applications.

### 3.6. Interplay Between Environmental Factors and Fruit Quality Traits: Insights from Key Mango Cultivars

To elucidate the relationships among climatic and geographical variables, cultivar, and fruit physicochemical attributes, Pearson correlation analyses results were visualized as a single chord diagram (Figure 5), and all significant pairwise correlations (*p* < 0.05) are listed in Supplementary Table S3. The nine environmental variables exhibited strong mutual correlations (e.g., Longitude-Elevation, r = −0.968, *p* < 0.001; Longitude-MAT, r = 0.927, *p* < 0.001; Latitude-T_min_, r = −0.822, *p* < 0.001), reflecting the tight covariation of climatic and geographical gradients across the sampled production regions. In contrast, only two environment–quality correlations reached nominal significance (*p* < 0.05): T_max_ was negatively correlated with carotene content (r = −0.332, *p* = 0.036) and positively correlated with moisture content (r = 0.327, *p* = 0.040). Neither of these correlations remained significant after Benjamini–Hochberg FDR correction (adjusted *p* = 0.98), indicating that environment–quality associations are weak and exploratory at the present sampling scale (Figure 5, Supplementary Table S3).

The strongest associations detected in the pooled network were those between cultivar type and quality traits: cultivar type was significantly correlated with total soluble solids (r = 0.598, *p* < 0.001), carotene (r = 0.535, *p* < 0.001), and pectin (r = 0.525, *p* < 0.001), and all three correlations remained significant after FDR correction (adjusted *p* < 0.01). Several quality traits also covaried strongly with one another (e.g., total acidity–TSS/TA ratio, r = −0.809, *p* < 0.001; crude protein–moisture, r = −0.711, *p* < 0.001; total dietary fiber–crude protein, r = 0.675, *p* < 0.001). Overall, the pooled correlation patterns indicate that fruit-quality variation is associated mainly with cultivar identity rather than with the sampled environmental gradients, underscoring the predominant role of genetic background in shaping mango fruit quality, and these correlations represent associations rather than causal relationships (Figure 5).

## 4. Discussion

### 4.1. Climatic Diversity and Regional Differentiation of Fruit Quality

The environmental determinants of fruit quality have been increasingly recognized as critical modulators acting across the pre- and post-harvest continuum [21]. The four mango-producing provinces examined in this study span a remarkable climatic gradient from the tropical maritime environment of Hainan (MAT 25.2 °C, RH 77%) to the high-altitude temperate conditions of Yunnan (elevation > 1400 masl, MAT 18.2 °C), representing one of the most diverse cultivation landscapes worldwide. This climatic diversity directly dictates the regional fruit quality profiles observed in this study. Hainan’s high-temperature and high-humidity regime promotes rapid fruit development and early harvest, consistent with its role as China’s earliest-season supplier. In contrast, Sichuan’s dry–hot valley climate (RH 63%, large diurnal temperature amplitude of 41.4 °C) favors sugar accumulation, as evidenced by the highest mean sucrose content (12.15 g/100 g) and TSS levels (18.5 °Brix) recorded in this region samples. Meanwhile Yunnan’s high-elevation and cool-temperature environment was associated with the highest carotene concentrations (mean 36.62 mg/100 g). This suggests that mild thermal stress may upregulate carotenoid biosynthesis as a photoprotective response, aligning with consistent with findings in other climacteric fruits [41]. These observations align with evidence that altitude-associated temperature and radiation gradients significantly modulate fruit morpho-physiological and biochemical attributes [10]. The pronounced climatic diversity necessitates region-specific cultivar deployment strategies [8], as cultivar performance is intimately linked to local hydrothermal conditions.

### 4.2. Environmental Drivers of Fruit Quality Traits

These environmental drivers exert profound influences on fruit quality traits, revealing the mechanistic linkages between regional climatic conditions and fruit physicochemical properties [15,30]. Our OPLS-DA identified relative humidity (VIP = 1.48) and extreme maximum temperature (VIP = 1.15) as the primary climatic discriminators among producing regions, exceeding the conventional VIP threshold of 1.0. These findings are consistent with the established role of vapor pressure deficit and temperature extremes in regulating fruit transpiration, photosynthate translocation, and secondary metabolite partitioning [15]. In the pooled correlation network (Figure 5), only two environment–quality associations reached nominal significance: extreme maximum temperature was negatively correlated with carotene content (r = −0.332, *p* = 0.036) and positively correlated with moisture content (r = 0.327, *p* = 0.040), and neither remained significant after Benjamini–Hochberg FDR correction. These environment–quality associations should therefore be interpreted as weak and exploratory at the present sampling scale. The negative T_max_-carotene association is nonetheless biologically plausible, because temperature extremes can influence carotenoid accumulation through effects on chloroplast development and carotenoid biosynthetic enzymatic activities [42,43] and gene expression [44]. In contrast, the strongest associations in the network were those between cultivar type and quality traits, such as total soluble solids (r = 0.598), carotene (r = 0.535), and pectin (r = 0.525), which were all robust to FDR correction (adjusted *p* < 0.01), reinforcing the conclusion that genetic background, rather than the sampled environmental gradients, is the predominant determinant of fruit quality. This pattern suggests that G × E interactions, widely documented in perennial fruit crops [30,45], manifest primarily through cultivar-dependent quality baselines rather than through strong universal environment–quality coupling.

Nevertheless, it is important to emphasize that the associations between environmental drivers and fruit quality traits discussed in this study were identified across spatially distinct orchards and supported by a validated, permutation-tested discriminant model. These associations are based on a limited number of per-cultivar accessions and represent associative rather than causal evidence. Furthermore, no correction for multiple comparisons was applied to the cultivar-specific correlation tests. Consequently, the inferred mechanistic links require further validation through dedicated, large-scale orchard sampling trials.

### 4.3. Cultivar Versus Geographic Origin: Relative Contributions to Quality Variation

A central finding of this study is that provincial origin alone failed to explain significant variation in any of the 12 measured quality traits (all ANOVA *p* > 0.05), whereas cultivar-specific profiling revealed pronounced differences across multiple parameters. For instance, *Tainong No. 1* consistently exhibited the highest TDF and IDF values, *Guire 82* excelled in TSS (up to 21.9 °Brix) and sugar content, and *Keitt* maintained a distinct low-TSS, high-acidity profile. These results indicate that genetic identity, rather than broad geographic provenance, is the predominant determinant of the fruit quality within the Chinese production system, a conclusion reinforced by the PCA results showing extensive inter-provincial overlap in quality profiles. However, the substantial within-cultivar variability observed, such as the 4.7-fold range in *Tainong No. 1* carotene content between Sichuan and Yunnan, and the fivefold glucose variation among Guangxi *Tainong No. 1* accessions, demonstrates that localized environmental conditions can profoundly modulate the expression of genetic potential. This G × E interactions is a well-established phenomenon in fruit tree physiology, where phenotypic plasticity allows cultivars to adapt metabolically to diverse growing conditions [21,46]. The Mantel-like correlation between geographic distance and quality dissimilarity (r = 0.059, *p* = 0.102) further supports the conclusion that spatial proximity alone is a poor predictor of quality similarity, likely because microclimate, soil characteristics, and orchard management practices operate at scales finer than provincial boundaries. This finding has important implications for geographic indication (GI) product certification and quality standardization in the Chinese mango industry [11,47].

### 4.4. Implications for Cultivar Deployment and Orchard Management

While cultivar fundamentally determines fruit quality potential [21], the pooled correlation patterns identified in this study offer practical guidance for implementing precision cultivation strategies. The strong, FDR-robust associations between cultivar type and key quality traits (total soluble solids, r = 0.598; carotene, r = 0.535; pectin, r = 0.525) indicate that cultivar selection is the primary lever for quality optimization. At the same time, the nominally significant negative association between extreme maximum temperature and carotene content (r = −0.332, *p* = 0.036), although exploratory, suggests that orchard siting should avoid regions with frequent extreme temperature maxima in order to protect carotenoid accumulation and fruit coloration. From a postharvest and processing perspective, the observed quality variation has direct industrial relevance [46]. *Tainong No. 1*’s intermediate and stable quality profile across regions makes it the most versatile cultivar for both fresh consumption and processing into dried products, purees, and juices. The high TSS and sugar content of *Guire 82* render it particularly suitable for premium fresh-market positioning. *Keitt*’s low TSS/TA ratio (2–5) and firm texture align with its established role as a late-season export cultivar with extended shelf-life potential. These cultivar–region matching recommendations are consistent with emerging frameworks for terroir-driven fruit quality optimization [18,48].

### 4.5. Limitations and Future Directions

Several limitations of this study warrant consideration. First, the sample size was uneven across provinces, with Yunnan represented by only 6 accessions, which may have limited statistical power for detecting provincial-level effects, particularly for high-variance traits such as carotene (*p* = 0.097). Second, the cross-sectional design captured fruit quality at a single harvest stage, whereas fruit maturation dynamics (modulated by temperature and photoperiod) may reveal additional environmental effects. Third, the environmental variables used represent macro-climatic averages; microclimate data (orchard-level temperature, soil moisture, and radiation) would provide finer resolution for dissecting G × E interactions. Fourth, the pooled correlation analysis (Figure 5) was based on 40 samples with nominal significance thresholds; the two environment–quality correlations detected did not survive Benjamini–Hochberg FDR correction and should be regarded as exploratory hypotheses to be tested in larger, multi-year datasets.

Future investigations should prioritize (i) multi-year, multi-harvest sampling designs to capture inter-annual climatic variability, (ii) metabolomics and transcriptomics approaches to elucidate the molecular mechanisms underlying environment-driven quality differentiation [7,49], (iii) comprehensive soil physicochemical characterization to disentangle edaphic from climatic effects, and (iv) integration of genomic prediction models to enable marker-assisted selection for region-specific quality traits [46]. In particular, within the context of metabolomics, UHPLC-MS metabolomics and altitudinal gradient approaches could be employed to elucidate the environmental determinants of bioactive compound (including phenolic compounds, flavonoids, and other health-related metabolites) accumulation in relation to cultivar altitude [50]. Collectively, such multi-dimensional approaches will be essential for developing climate-resilient fruit production systems in an era of accelerating environmental change. Fruits from different geographical origins exhibit significant differences in their flavor profiles. VOCs analysis can elucidate the causes underlying these distinctive sensory characteristics [42]. Future research should further apply VOCs profiling to gain a more comprehensive understanding of how geography and climate influence overall flavor from nutritional composition to sensory acceptability [51]. This knowledge would not only enhance our understanding of fruit quality but also support the development of climate-resilient, processing-oriented crop systems.

## 5. Conclusions

This study provides a comprehensive, multi-dimensional characterization of mango fruit quality across four principal production provinces in China, integrating 12 physicochemical traits, 9 environmental variables, and 40 groups representing 6 commercially predominant cultivars. Our integrated analytical framework (combining PCA, OPLS-DA with VIP scoring, hierarchical clustering, Pearson correlation analysis) suggests that cultivar, rather than provincial origin, appears to be the predominant determinant of fruit quality. This is supported by the absence of significant provincial-level differences for any measured trait (*p* > 0.05) and the extensive PCA score overlap among regions. Crucially, the pooled correlation network (Figure 5) showed that fruit-quality variation was associated mainly with cultivar identity, such as cultivar type correlated significantly with total soluble solids (r = 0.598), carotene (r = 0.535), and pectin (r = 0.525), all robust to FDR correction—whereas environment–quality associations were weak and exploratory, with only two nominally significant correlations (T_max_–carotene, r = −0.332; T_max_–moisture, r = 0.327) that did not survive multiple-testing correction. As summarized in Figure 6, cultivar appears to set the primary quality potential of the fruit, whereas geographic and climatic conditions modulate the expression of quality traits through cultivar-specific G × E interactions. These findings provide a basis for an evidence-based framework for cultivar-region matching in production, with direct applications in precision orchard characterization and quality-oriented cultivation management. Furthermore, these insights offer practical implications for the processing industry, particularly in selecting cultivars for fresh-market or processing. Consequently, integrating these G × E interaction patterns into industrial supply chains could contribute to improve the standardization and nutritional consistency of processed mango products. As climate conditions continue to alter temperature and precipitation regimes, the cultivar-specific environment–quality relationships characterized herein will become increasingly valuable for guiding adaptive cultivation strategies and safeguarding the nutritional and sensory quality of this economically vital fruit crop.

## Figures and Tables

**Figure 1:**
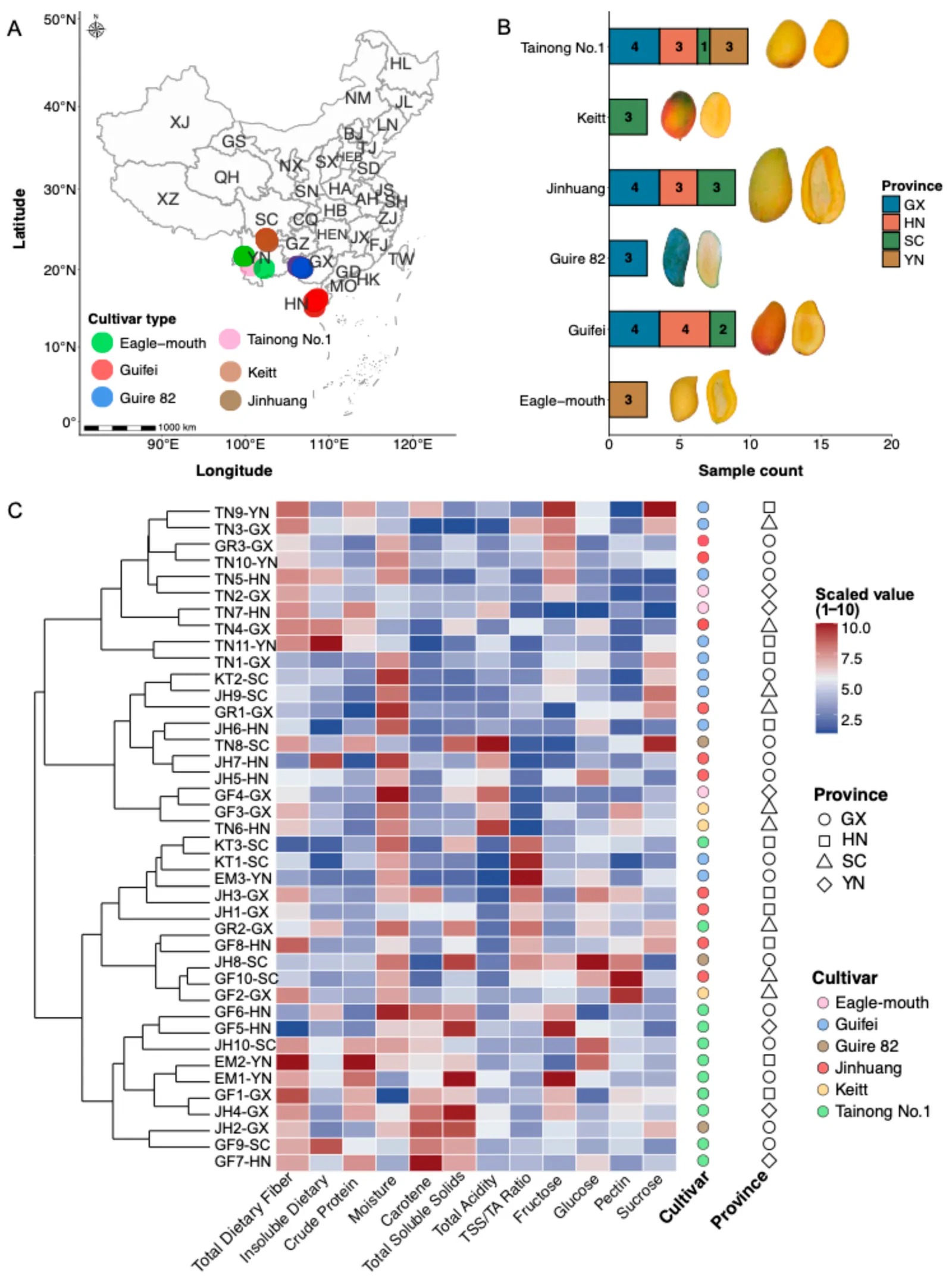
Geographical distribution of sampling sites and morphological characteristics of six mango cultivars, along with the quality trait profiles and hierarchical clustering analysis. (**A**) Map illustrating the sampling locations of different varieties across major cultivation regions in China. (**B**) Bar chart showing the sample size distribution across six cultivars (*EM*, *Eagle-mouth*; *GF*, *Guifei*; *GR*, *Guire 82*; *JH*, *Jinhuang*; *KT*, *Keitt*; *and TN*, *Tainong No. 1*), with representative fruit images. (**C**) Dendrogram of hierarchical clustering analysis (Ward’s method) and heatmap visualizing the normalized (Z-score: −10) quality trait profiles of 40 individual samples from various provinces (GX, Guangxi; HN, Hainan; SC, Sichuan; YN, Yunnan).

**Figure 2:**
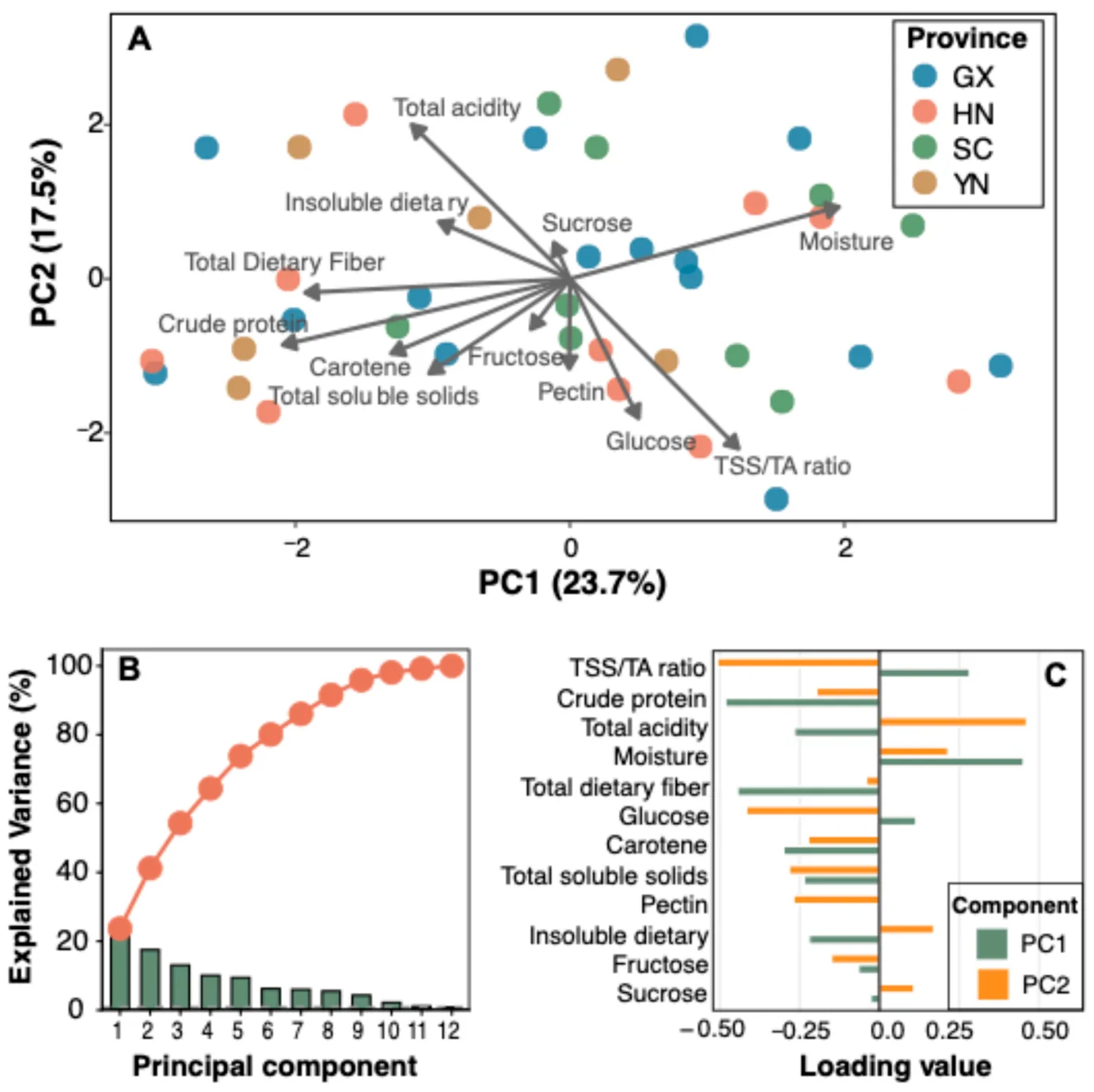
Principal component analysis (PCA) of fruit quality parameters across four geographical origins. (**A**) PCA biplot displaying the distribution of samples from Guangxi, Hainan, Sichuan, and Yunnan, with vectors indicating the contributions of 12 quality traits to the first two principal components (PC1 and PC2). (**B**) Scree plot illustrating the explained variance ratio of each principal component. (**C**) Loading plot presenting the contributions (loadings) of individual quality traits to PC1 and PC2, identifying moisture, crude protein, and total dietary fiber as the most influential parameters.

**Figure 3:**
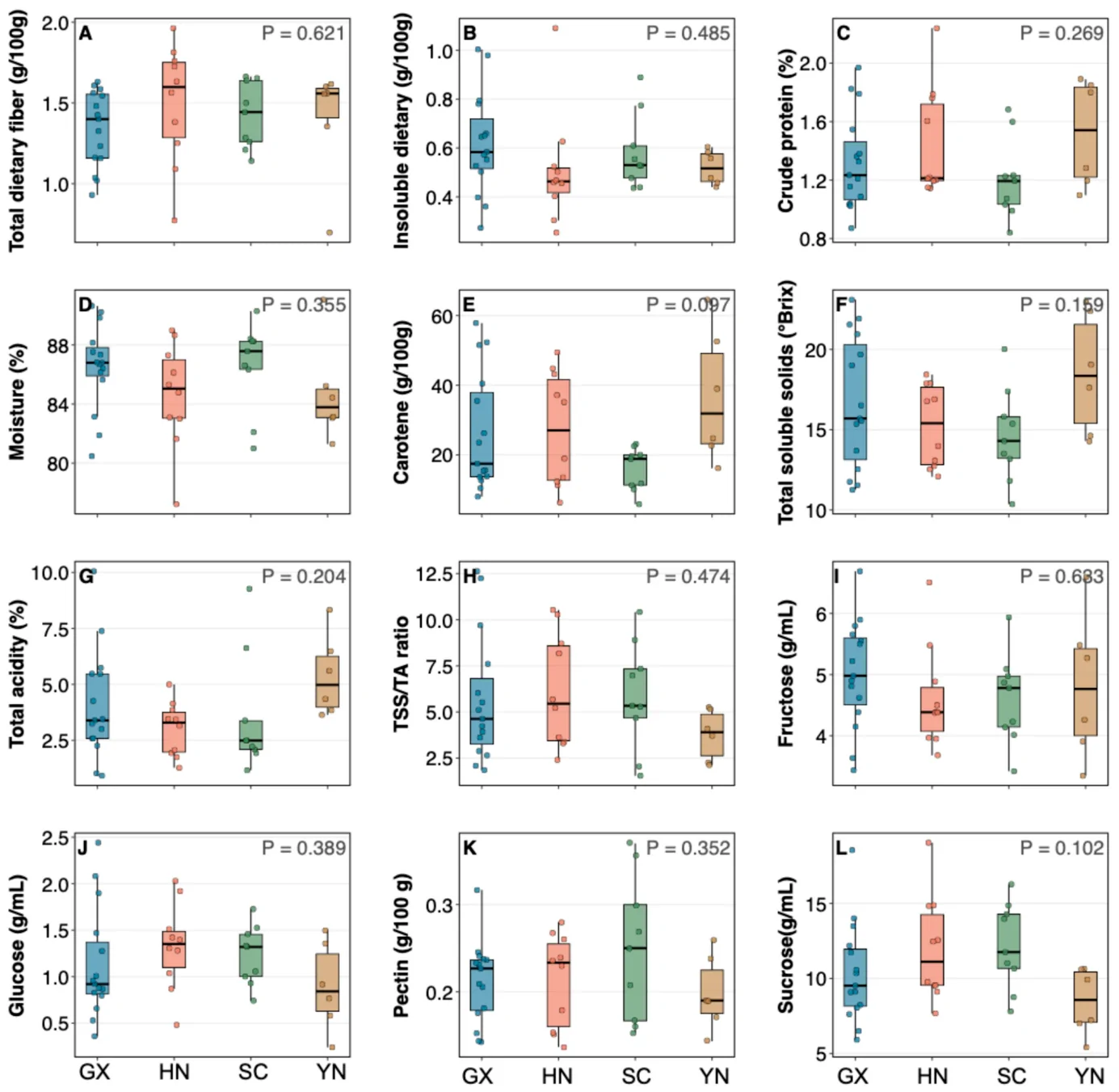
Comparison of fruit quality traits across four provinces (Guangxi, Hainan, Sichuan, and Yunnan). The 12 panels represent the variation in nutritional and physicochemical parameters. (**A**) total dietary fiber; (**B**) insoluble dietary fiber; (**C**) crude protein; (**D**) moisture; (**E**) carotene; (**F**) total soluble solids; (**G**) total acidity; (**H**) TSS/TA ratio; (**I**) fructose; (**J**) glucose; (**K**) pectin; (**L**) sucrose. Within each boxplot, the central line represents the median, while the box indicates the interquartile range. The *p*-values shown in the upper right corners indicate the significance level of the regional differences based on one-way ANOVA.

**Figure 5:**
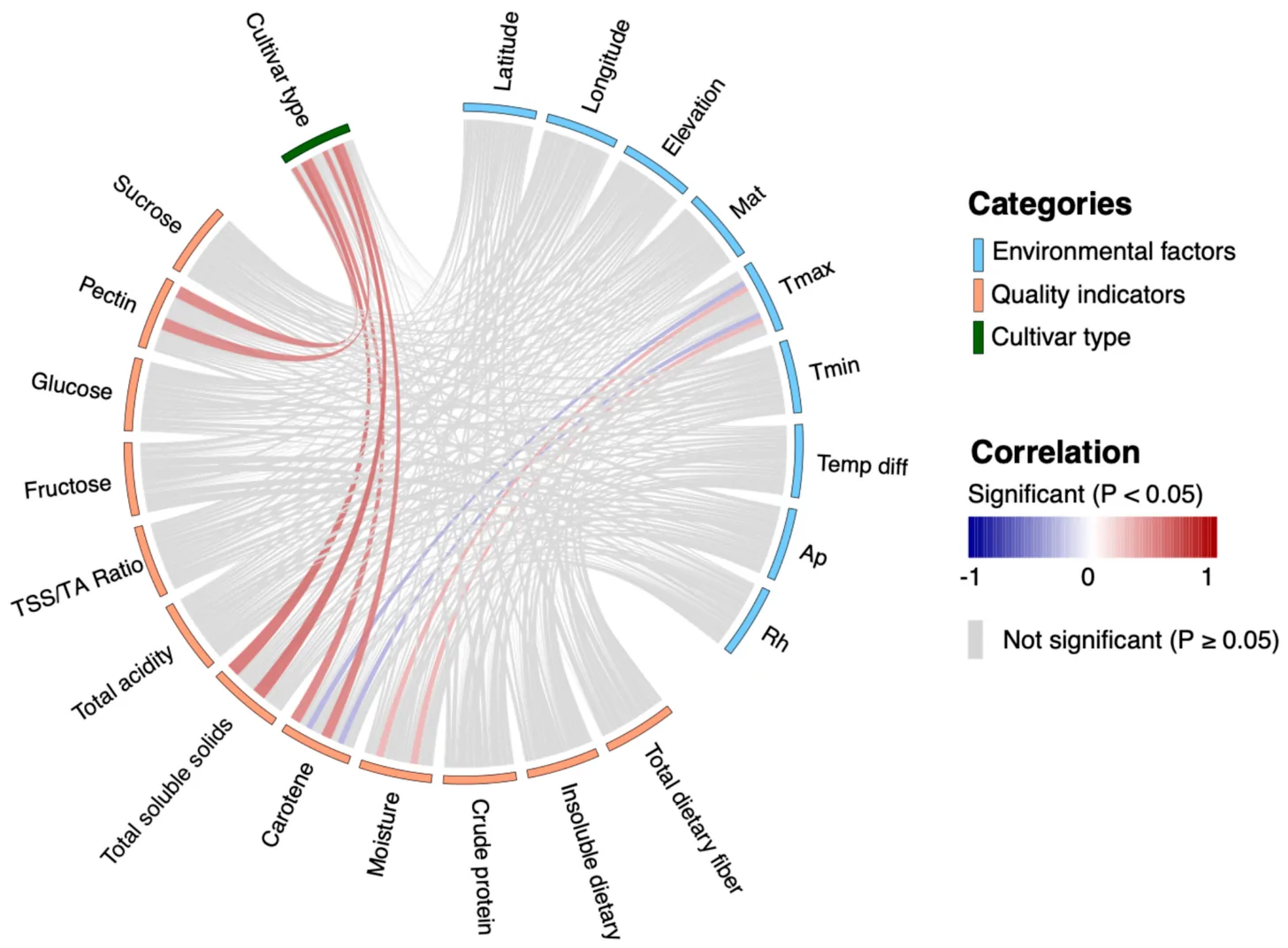
Pooled correlation chord diagram among environmental factors, fruit quality traits, and cultivar type across all 40 mango samples. The chord diagram visualizes Pearson correlations between nine environmental variables (blue grid segments: Latitude, Longitude, Elevation, MAT, T_max_, T_min_, ΔT, AP, RH) and twelve quality traits (orange grid segments: total dietary fiber, insoluble dietary fiber, crude protein, moisture, carotene, TSS, TA, TSS/TA ratio, fructose, glucose, pectin, sucrose). Chord width is scaled to the absolute correlation coefficient (|r|); red and blue chords represent significant positive and negative correlations, respectively (*p* < 0.05), while grey chords indicate non-significant associations (*p* ≥ 0.05).

**Figure 6:**
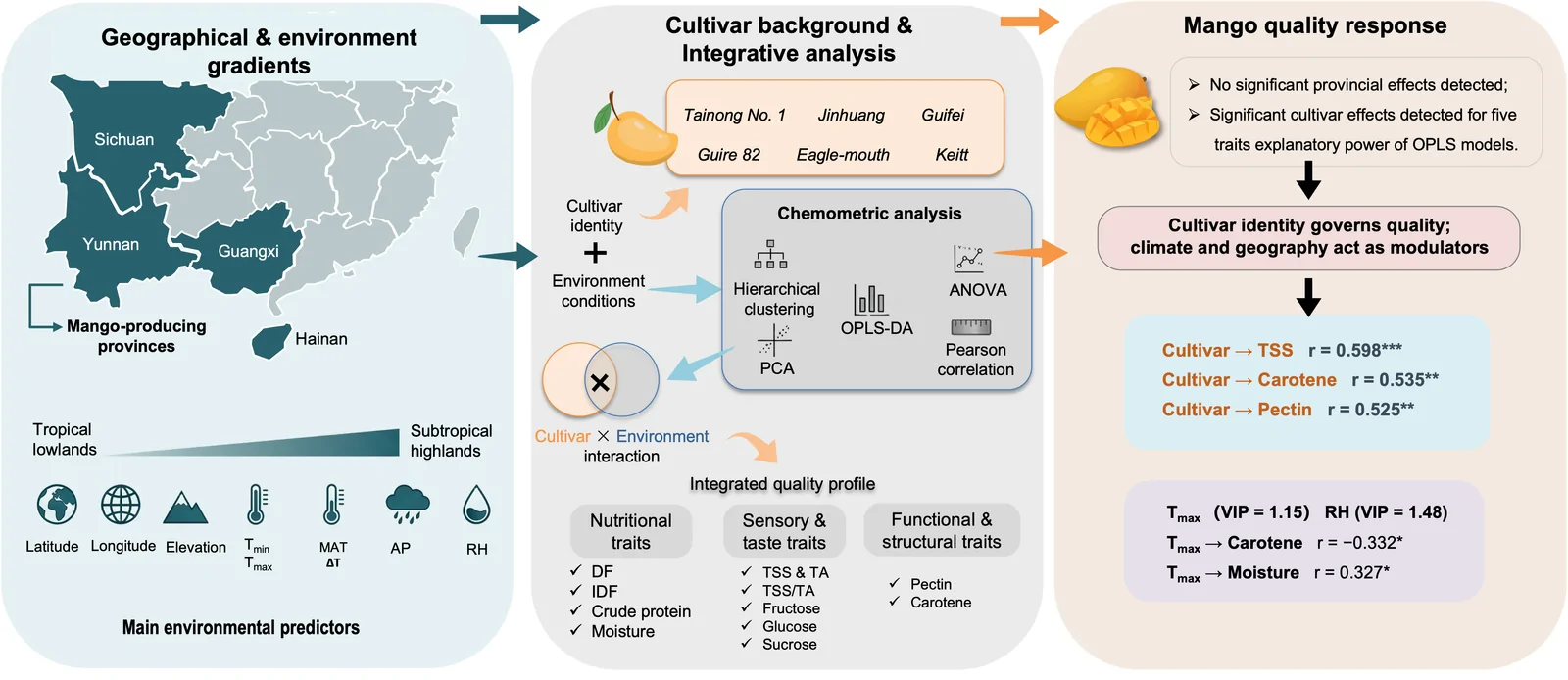
Potential mechanism of environmental and cultivar determinants of mango fruit quality. Geographic origin, climatic factors, and cultivar converge through genotype × environment interactions to modulate twelve physicochemical quality attributes. VIP scores denote variable importance from OPLS-DA; correlation coefficients (r) indicate significant Pearson associations (*p* < 0.05). RH, mean relative humidity; T_max_, extreme minimum temperature; T_min_, extreme maximum temperature; MAT, mean annual temperature; ΔT, temperature difference; AP, annual precipitation; TDF, total dietary fiber; IDF, insoluble dietary fiber; TSS, total soluble solids; TA, total acidity. Asterisks represent the significant difference (* *p *< 0.05, ** *p *< 0.01, *** *p *< 0.001).

**Figure A1:**
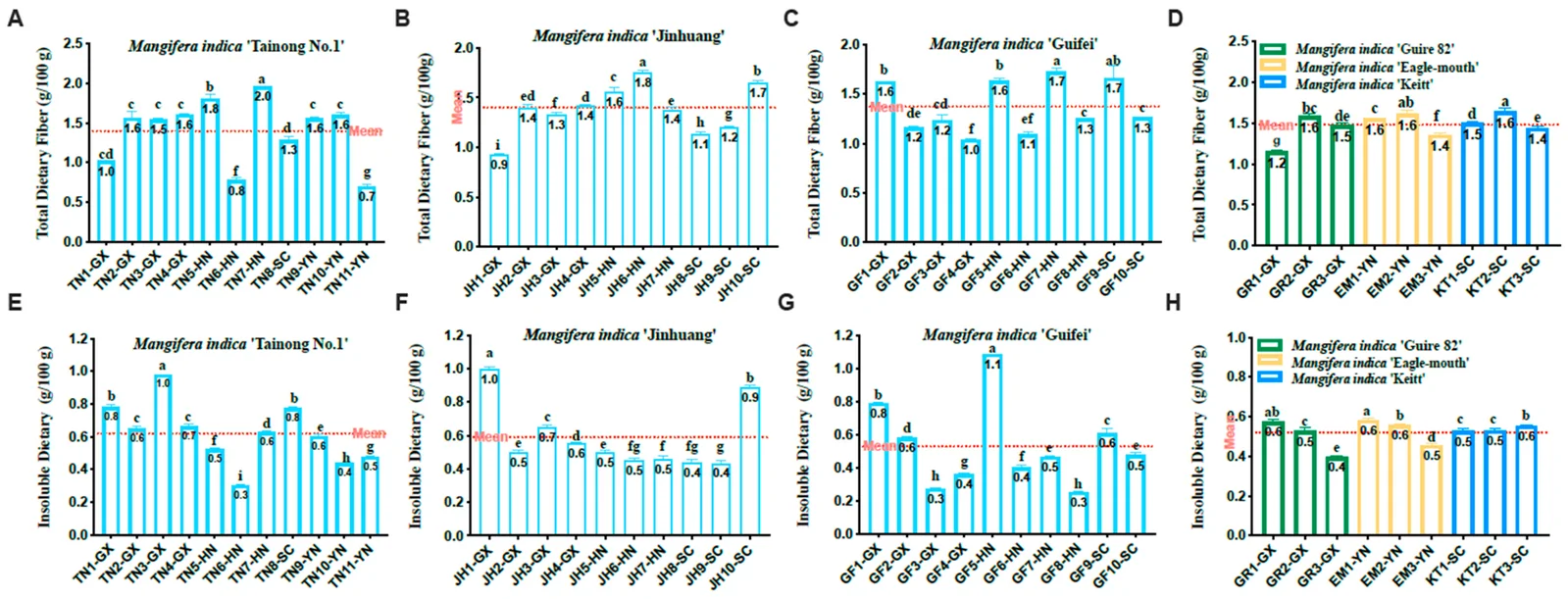
Comparison of (**A**–**D**) total dietary fiber and (**E**–**H**) insoluble dietary fiber contents in fruit across four producing provinces (Guangxi, Hainan, Sichuan, and Yunnan). Data are presented as means ± SD (n = 3). Different letters indicate significant differences at *p* < 0.05 (Tukey’s HSD test). The suffix of sample ID denotes the province of origin: GX, Guangxi; HN, Hainan; SC, Sichuan; YN, Yunnan.

**Figure A2:**
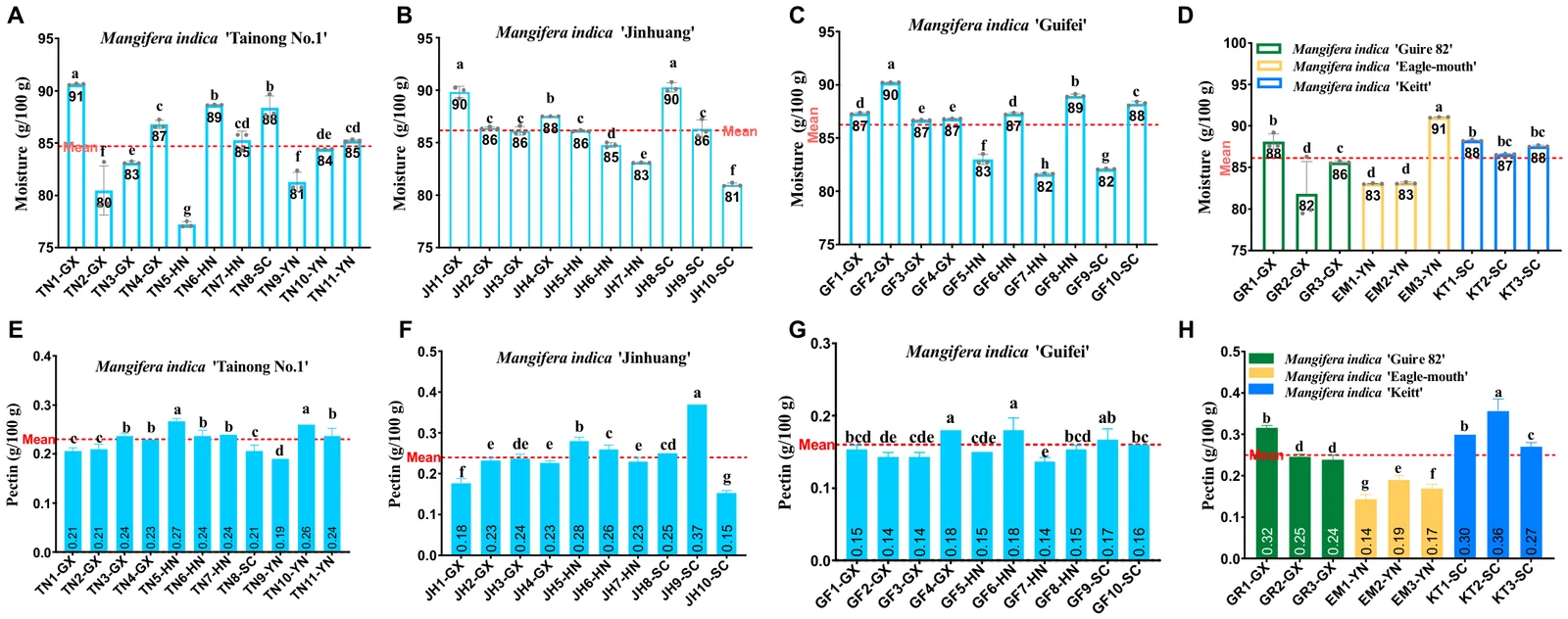
Comparison of (**A**–**D**) moisture and (**E**–**H**) pectin contents in fruit across four producing provinces. Data are presented as means ± SD (n = 3). Different letters indicate significant differences at *p* < 0.05 (Tukey’s HSD test). The suffix of sample ID denotes the province of origin: GX, Guangxi; HN, Hainan; SC, Sichuan; YN, Yunnan.

**Figure A3:**
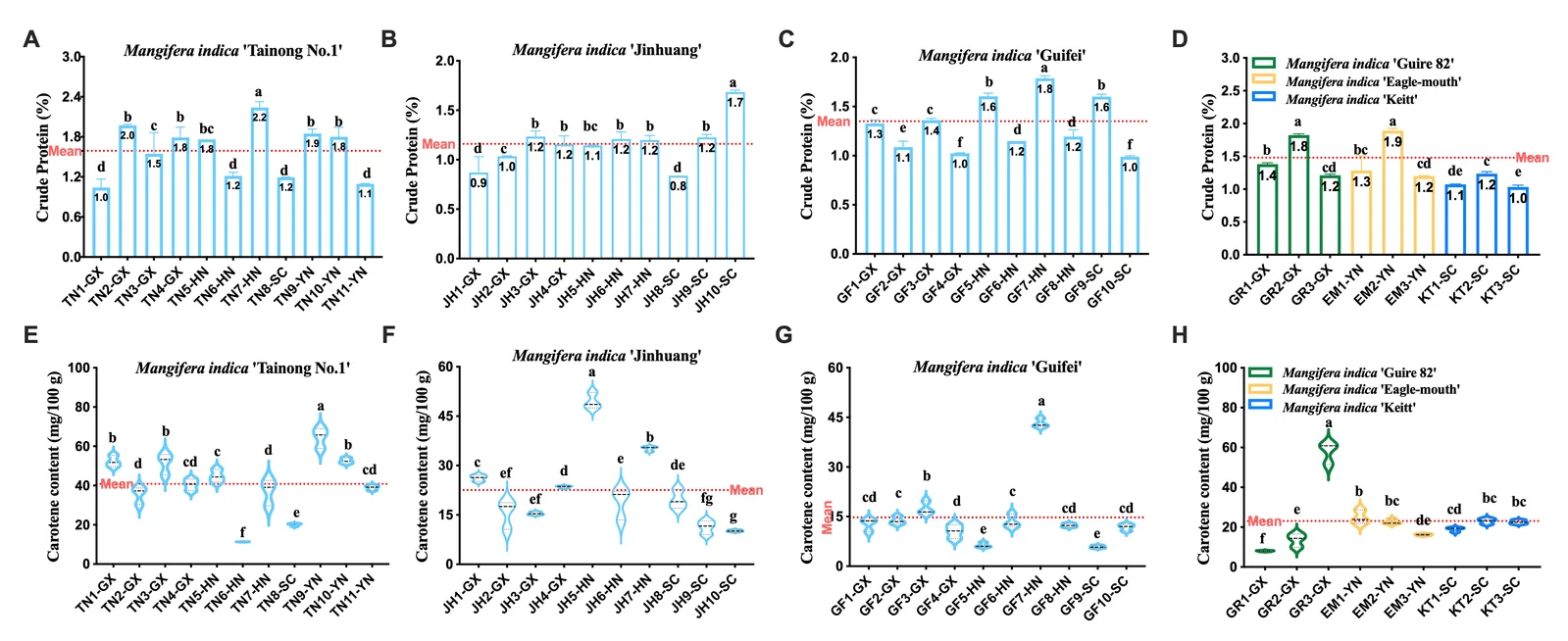
Comparison of (**A**–**D**) crude protein and (**E**–**H**) carotene contents in mango fruits across four producing provinces. Data are presented as means ± SD (n = 3). Different letters indicate significant differences at *p* < 0.05 (Tukey’s HSD test). The suffix of sample ID denotes the province of origin: GX, Guangxi; HN, Hainan; SC, Sichuan; YN, Yunnan.

**Figure A4:**
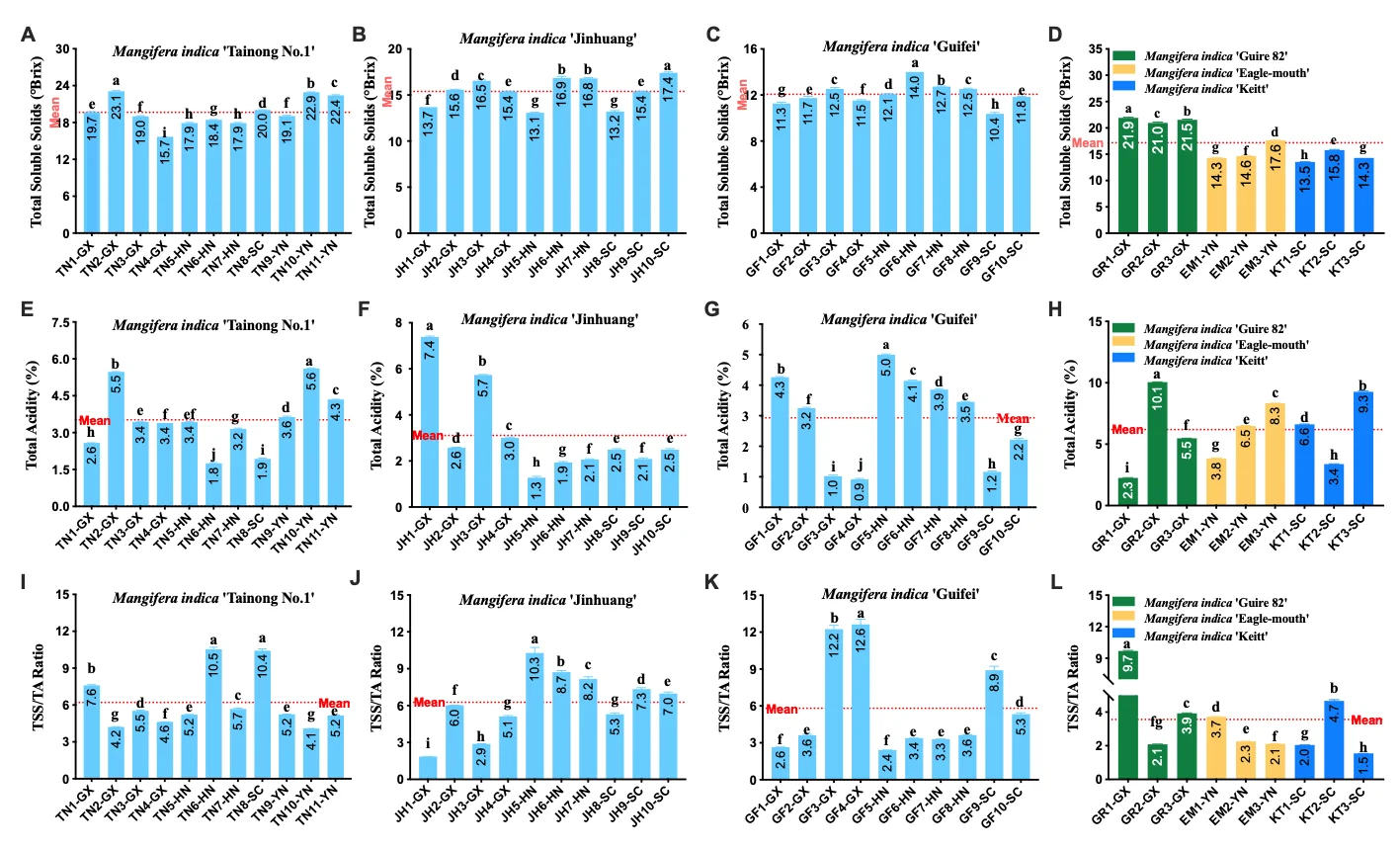
Comparison of (**A**–**D**) total soluble solids (TSS), (**E**–**H**) total acidity (TA), and (**I**–**L**) TSS/TA ratio in fruit across four producing provinces. Data are presented as means ± SD (n = 3). Different letters indicate significant differences at *p* < 0.05 (Tukey’s HSD test). The suffix of sample ID denotes the province of origin: GX, Guangxi; HN, Hainan; SC, Sichuan; YN, Yunnan.

**Figure A5:**
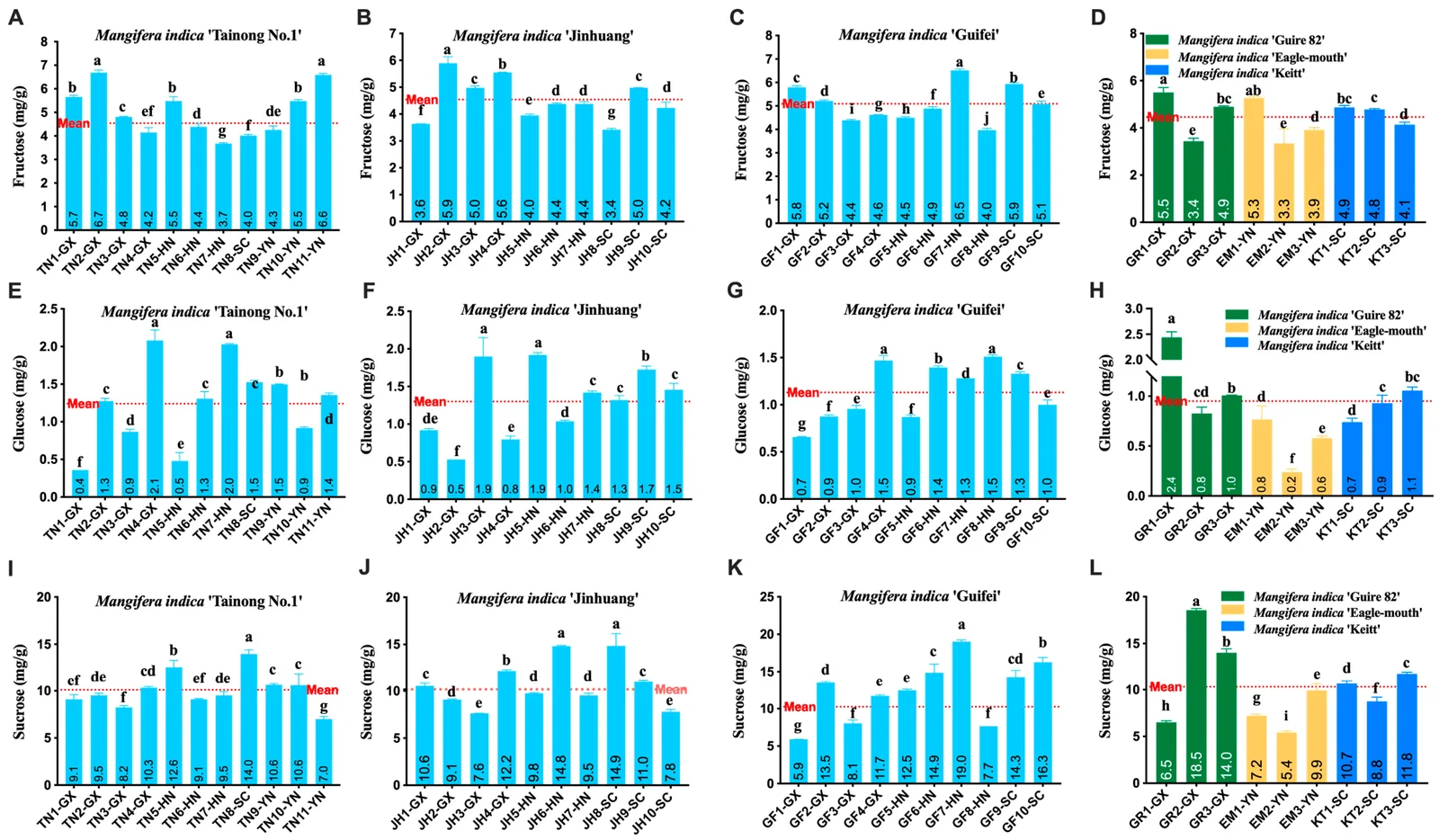
Comparison of (**A**–**D**) fructose, (**E**–**H**) glucose and (**I**–**L**) sucrose contents in fruit across four producing provinces. Data are presented as means ± SD (n = 3). Different letters indicate significant differences at *p* < 0.05 (Tukey’s HSD test). The suffix of sample ID denotes the province of origin: GX, Guangxi; HN, Hainan; SC, Sichuan; YN, Yunnan.

## Data Availability

The original contributions presented in the study are included in the article/Supplementary Material, further inquiries can be directed to the corresponding authors.

## Supplementary Materials

The following supporting information can be downloaded at: https://www.mdpi.com/article/10.3390/foods15193425/s1; Table S1. OPLS-DA model performance and validation statistics; Table S2. Model validation by class-label permutation test (n = 200); Table S3. Pairwise Pearson correlations among environmental factors, fruit quality traits, and cultivar type that remained significant after Benjamini–Hochberg FDR correction (FDR-adjusted *p* < 0.05) across all 40 mango samples.

## Abbreviations

The following abbreviations are used in this manuscript:

AP	Annual Precipitation
CMA	China Meteorological Administration
EM	Eagle-Mouth (Mango Cultivar)
FW	Fresh Weight
G × E	Genotype × Environment
GF	Guifei (Mango Cultivar)
GI	Geographic Indication
GR	Guire 82 (Mango Cultivar)
GX	Guangxi (Province)
HN	Hainan (Province)
HPLC	High-Performance Liquid Chromatography
HSD	Honestly Significant Difference
IDF	Insoluble Dietary Fiber
JH	Jinhuang (Mango Cultivar)
KT	Keitt (Mango Cultivar)
masl	Meters above sea level
MAT	Mean Annual Temperature
OPLS-DA	Orthogonal Partial Least Squares Discriminant Analysis
PCs	Principal Components
RH	Mean Relative Humidity
SC	Sichuan (Province)
SE	Standard Errors
TA	Total Acidity
TDF	Total Dietary Fiber
T_max_	Extreme Maximum Temperature
T_min_	Extreme Minimum Temperature
TN	Tainong No. 1 (Mango Cultivar)
TSS	Total Soluble Solids
VIP	Variable Importance in Projection
VOCs	Volatile Organic Compound
YN	Yunnan (Province)
ΔT	Temperature Difference (T_max_ − T_min_)
