# Supplementary material for: Geographical and Climatic Effects on the Quality Attributes of Chinese Mango Fruit
Source: Foods. 2026 Sep 24;15(19):3425. doi: 10.3390/foods15193425 (PMC13664513; doi:10.3390/foods15193425)
Supplement: Supplementary file 1 [file foods-15-03425-s001.zip › foods-4489054-supplementary.pdf]

Supplementary Table S1. OPLS-DA model performance and validation statistics.

Predictive component	R2X(cum)	R2Y(cum)	Q2(cum)
1	0.626	0.311	0.293
2	0.854	0.546	0.508
Model (3 components)	0.921	0.798	0.764

Note: *R2X*(cum)/*R2Y*(cum): cumulative fractions of predictor and class-response variance explained; Q2(cum): cross-validated predictive ability.

Supplementary Table S2. Model validation by class-label permutation test (n = 200).

Permutation statistic	Observed model	Permuted models	Empirical p-value
R2Y(cum)	0.798	0.0365–0.230	0.005
Q2(cum)	0.764	−0.448 to +0.030	0.005
R2X(cum)	0.921	0.767–0.927	–

Note: Permutation test with 200 random permutations of class labels to assess model significance and overfitting risk.

Supplementary Table S3. Pairwise Pearson correlations among environmental factors, fruit quality traits, and cultivar type that remained significant after Benjamini–Hochberg FDR correction (FDR-adjusted P < 0.05) across all 40 mango samples.

Variable 1	Variable 2	Category	Correlation (r)	P value	FDR-adjusted P	Significance
Longitude	Elevation	Env × Env	-0.968	< 0.001	< 0.001	***
Longitude	Mat	Env × Env	0.927	< 0.001	< 0.001	***
Elevation	Mat	Env × Env	-0.918	< 0.001	< 0.001	***
T_{min}	Temp diff	Env × Env	-0.876	< 0.001	< 0.001	***
Latitude	Tmin	Env × Env	-0.822	< 0.001	< 0.001	***
Total acidity	TSS/TA ratio	Quality × Quality	-0.809	< 0.001	< 0.001	***
Elevation	Rh	Env × Env	-0.795	< 0.001	< 0.001	***
Latitude	Mat	Env × Env	-0.791	< 0.001	< 0.001	***
Latitude	Rh	Env × Env	-0.790	< 0.001	< 0.001	***
Longitude	Rh	Env × Env	0.766	< 0.001	< 0.001	***
Latitude	Longitude	Env × Env	-0.765	< 0.001	< 0.001	***
Mat	T_{min}	Env × Env	0.754	< 0.001	< 0.001	***
Latitude	Temp diff	Env × Env	0.741	< 0.001	< 0.001	***
Crude protein	Moisture	Quality × Quality	-0.711	< 0.001	< 0.001	***
T_{max}	Temp diff	Env × Env	0.707	< 0.001	< 0.001	***
Longitude	T_{min}	Env × Env	0.696	< 0.001	< 0.001	***
Latitude	Elevation	Env × Env	0.692	< 0.001	< 0.001	***
Total dietary fiber	Crude protein	Quality × Quality	0.675	< 0.001	< 0.001	***
Mat	Rh	Env × Env	0.653	< 0.001	< 0.001	***
Latitude	Ap	Env × Env	-0.647	< 0.001	< 0.001	***
Longitude	Ap	Env × Env	0.632	< 0.001	< 0.001	***
Total dietary fiber	Moisture	Quality × Quality	-0.612	< 0.001	< 0.001	***
Elevation	T_{min}	Env × Env	-0.605	< 0.001	< 0.001	***
Total soluble solids	Cultivar type	Quality × Cultivar	0.598	< 0.001	< 0.001	***
Mat	Ap	Env × Env	0.586	< 0.001	< 0.001	***
T_{min}	Ap	Env × Env	0.552	< 0.001	0.002	**
Carotene	Cultivar type	Quality × Cultivar	0.535	< 0.001	0.0032	**
T_{min}	Rh	Env × Env	0.528	< 0.001	0.0038	**

Variable 1	Variable 2	Category	Correlation (r)	P value	FDR-adjusted P	Significance
Pectin	Cultivar type	Quality × Cultivar	0.525	< 0.001	0.004	**
Elevation	Ap	Env × Env	-0.493	0.0012	0.0094	**
Carotene	Total soluble solids	Quality × Quality	0.474	0.002	0.015	*
Ap	Rh	Env × Env	0.433	0.0053	0.0382	*
Temp diff	Rh	Env × Env	-0.420	0.0069	0.0485	*

Note: Significance levels are based on FDR-adjusted P-values: *, P < 0.05; **, P < 0.01; ***, P < 0.001. FDR-adjusted P-values listed as < 0.001 correspond to values rounded to 0. No environment × quality correlation survived FDR correction. Mat, mean annual temperature; Tmax, extreme maximum temperature; Tmin, extreme minimum temperature; Temp diff, temperature difference; Ap, annual precipitation; Rh, mean relative humidity.
